# Supplementary material for: Mitochondrial RNA cytosolic leakage drives the SASP
Source: Nat Commun. 2025 Dec 15;16:10992. doi: 10.1038/s41467-025-66159-z (PMC12705736; doi:10.1038/s41467-025-66159-z)
Supplement: Supplementary file 1 — Supplementary Information [file 41467_2025_66159_MOESM1_ESM.pdf]

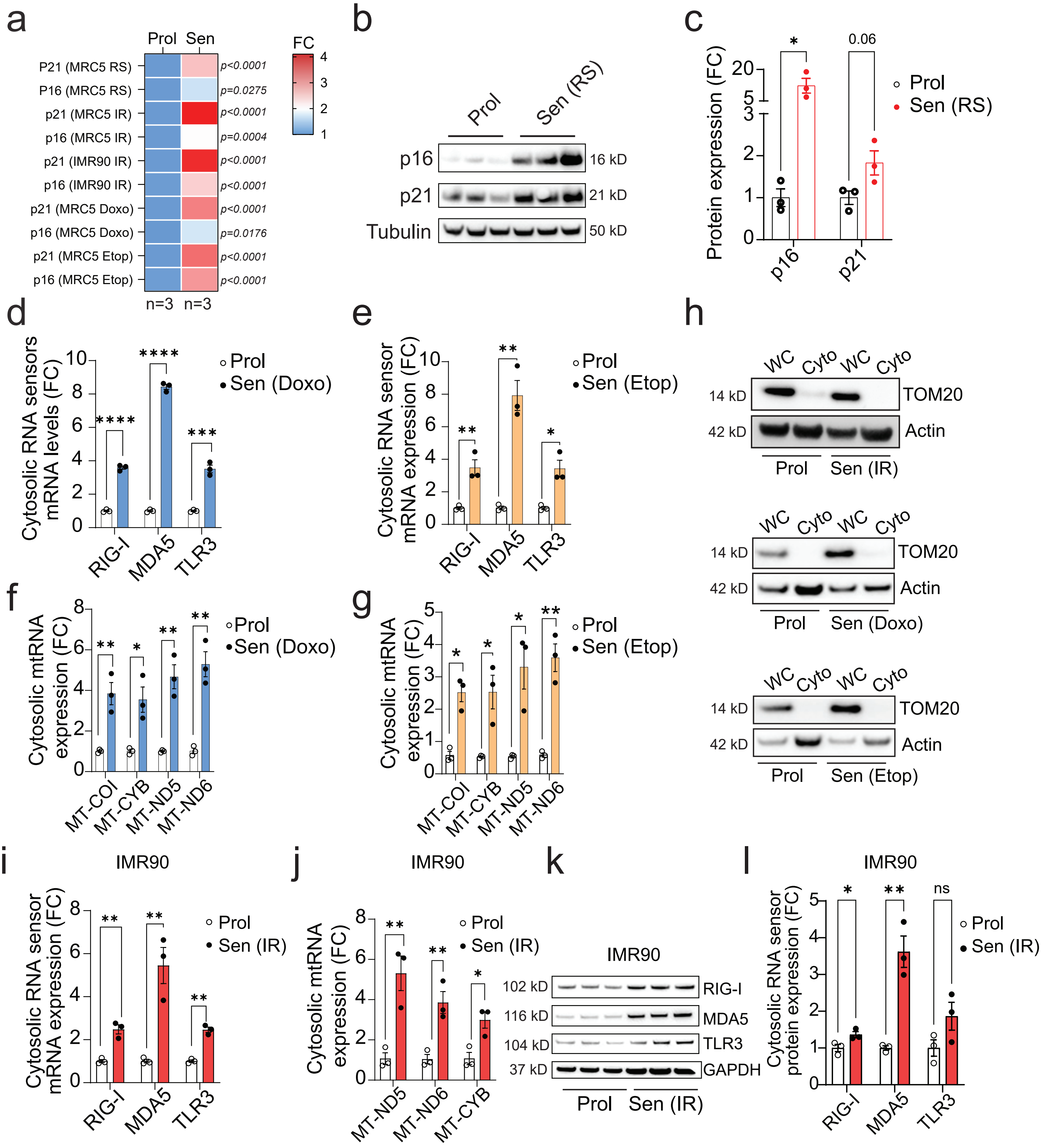
a
Prol Sen
FC
P21 (MRC5 RS) p<0.0001
P16 (MRC5 RS) p=0.0275
p21 (MRC5 IR) p<0.0001
p16 (MRC5 IR) p=0.0004
p21 (IMR90 IR) p<0.0001
p16 (IMR90 IR) p<0.0001
p21 (MRC5 Doxo) p<0.0001
p16 (MRC5 Doxo) p=0.0176
p21 (MRC5 Etop) p<0.0001
p16 (MRC5 Etop) p<0.0001
n=3 n=3
b
Prol
Sen (RS)
p16 16 kD
p21 21 kD
Tubulin 50 kD
c
Protein expression (FC)
0.06
Prol
Sen (RS)
p16
p21
d
Cytosolic RNA sensors mRNA levels (FC)
Prol
Sen (Doxo)
RIG-I
MDA5
TLR3
e
Cytosolic RNA sensor mRNA expression (FC)
Prol
Sen (Etop)
RIG-I
MDA5
TLR3
h
WC Cyto WC Cyto
14 kD TOM20
42 kD Actin
Prol Sen (IR)
14 kD TOM20
42 kD Actin
Prol Sen (Doxo)
14 kD TOM20
42 kD Actin
Prol Sen (Etop)
f
Cytosolic mtRNA expression (FC)
Prol
Sen (Doxo)
MT-COI
MT-CYB
MT-ND5
MT-ND6
g
Cytosolic mtRNA expression (FC)
Prol
Sen (Etop)
MT-COI
MT-CYB
MT-ND5
MT-ND6
i
IMR90
Cytosolic RNA sensor mRNA expression (FC)
Prol
Sen (IR)
RIG-I
MDA5
TLR3
j
IMR90
Cytosolic mtRNA expression (FC)
Prol
Sen (IR)
MT-ND5
MT-ND6
MT-CYB
k
IMR90
102 kD RIG-I
116 kD MDA5
104 kD TLR3
37 kD GAPDH
Prol Sen (IR)
l
IMR90
Cytosolic RNA sensor protein expression (FC)
ns
Prol
Sen (IR)
RIG-I
MDA5
TLR3

Supplementary Figure 1 - Cytosolic mtRNA leakage occurs irrespectively of cell type and senescence-inducing stimulus. (a) Heatmap showing mRNA expression of senescence markers p16 and p21 across different models of senescence in MRC5 and IMR90 human fibroblasts, including replicative senescence (RS), ionizing radiation (IR), doxorubicin (Doxo), and etoposide (Etop). n = 3 independent experiments per condition. **(b)** Representative Western blot of p16 and p21 protein levels in proliferating and replicatively senescent (RS) MRC5 cells. n=3 independent experiments. **(c)** Quantification of Western blot in (b), showing fold change in p16 and p21 expression in senescent cells relative to proliferating controls. n = 3 per group. **(d, e)** qPCR quantification of cytosolic RNA sensor mRNA levels (RIG-I, MDA5, and TLR3) in proliferating and senescent MRC5 fibroblasts treated with (d) doxorubicin or (e) etoposide. n=3 independent experiments. **(f, g)** qPCR quantification of cytosolic mitochondrial RNA transcripts (MT-CO1, MT-CYB, MT-ND5, MT-ND6) in proliferating and senescent MRC5 cells treated with (f) doxorubicin or (g) etoposide. n=3 independent experiments. **(h)** Western blot for TOM20 and Actin in whole-cell (WC) and cytosolic (Cyto) fractions from proliferating and senescent MRC5 fibroblasts treated with IR, doxorubicin, or etoposide, confirming purity of cytosolic fractions. Representative blot of n=3 independent experiments. **(i)** qPCR analysis of RNA sensor expression (RIG-I, MDA5, TLR3) in proliferating and IR-induced senescent IMR90 fibroblasts. n = 3 independent experiments. **(j)** qPCR quantification of cytosolic mitochondrial transcripts (*MT-ND5, MT-ND6, MT-CYB*) in proliferating and IR-induced senescent IMR90 cells. n = 3 independent experiments. **(k)** Representative Western blot showing increased protein levels of RIG-I, MDA5, and TLR3 in senescent (IR) IMR90 fibroblasts compared to proliferating controls. **(l)** Quantification of Western blot in (k), showing fold change in RIG-I, MDA5 and TLR3 expression in senescent cells relative to proliferating controls. n = 3 *per* group. Data are shown as mean ± s.e.m. Statistical significance was assessed using two-sided unpaired Student's *t*-test. **(c)** p=0.0334, p=0.06; **(d)** p<0.0001, p<0.0001, p=0.0004; **(e)** p=0.0076, p=0.0017, p=0.0101; **(f)** p=0.0067, p=0.0157, p=0.0034, p=0.0025; **(g)** p=0.0165, p=0.0342, p=0.0154, p=0.0034; **(i)** p=0.0028, p=0.0062, p=0.0016; **(j)** p=0.0094, p=0.0088, p=0.0197; **(l)** p=0.0112, p=0.0023, p=0.1021.

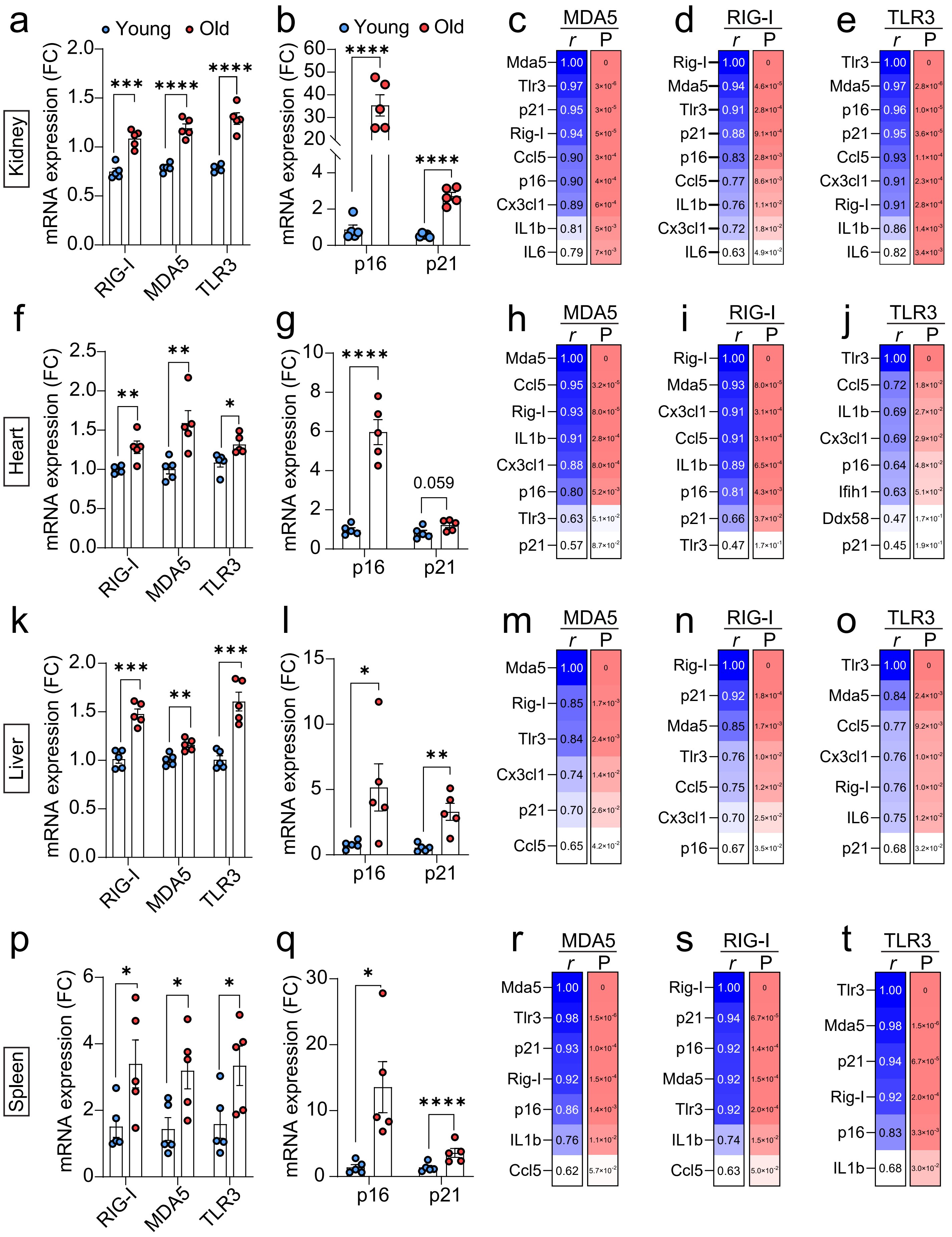

a b c d e
Young Old
Kidney
mRNA expression (FC)
RIG-I MDA5 TLR3
p16 p21
MDA5 RIG-I TLR3
r P
f g h i j
Heart
0.059
k l m n o
Liver
p q r s t
Spleen

Supplementary Figure 2 - Expression of cytosolic RNA sensors correlates with senescence markers in different tissues during aging. mRNA expression levels of **(a, f, k, p)** cytosolic RNA sensors and **(b, g, l, q)** p16 and p21 in the kidney, heart, liver and spleen, respectively, of young and old mice. The correlation coefficient between expression levels of **(c, h, m, r)** MDA5, **(d, i, n, s)** RIG-I and **(e, j, o, t)** TLR3 and different senescence markers in the kidney, heart, liver and spleen, respectively, of young and old animals. n=5 animals per group. Data are mean ± s.e.m. Statistical significance was assessed using a two-sided Student's unpaired t-test **(a, b, f, g, k, l, p** and **q)** and Pearson's correlation coefficient **(c-e, h-j, m-o, r-t)**. **(a)** $p=0.002$, $p<0.0001$, $p<0.0001$; **(b)** $p<0.0001$, $p<0.0001$; **(f)** $p=0.0077$, $p=0.009$, $p=0.0171$; **(g)** $p<0.0001$, $p=0.059$; **(k)** $p=0.0001$, $p=0.0039$, $p=0.0004$; **(l)** $p=0.0421$, $p=0.0033$; **(p)** $p=0.0433$, $p=0.0262$, $p=0.0432$; **(q)** $p=0.0143$, $p<0.0001$.

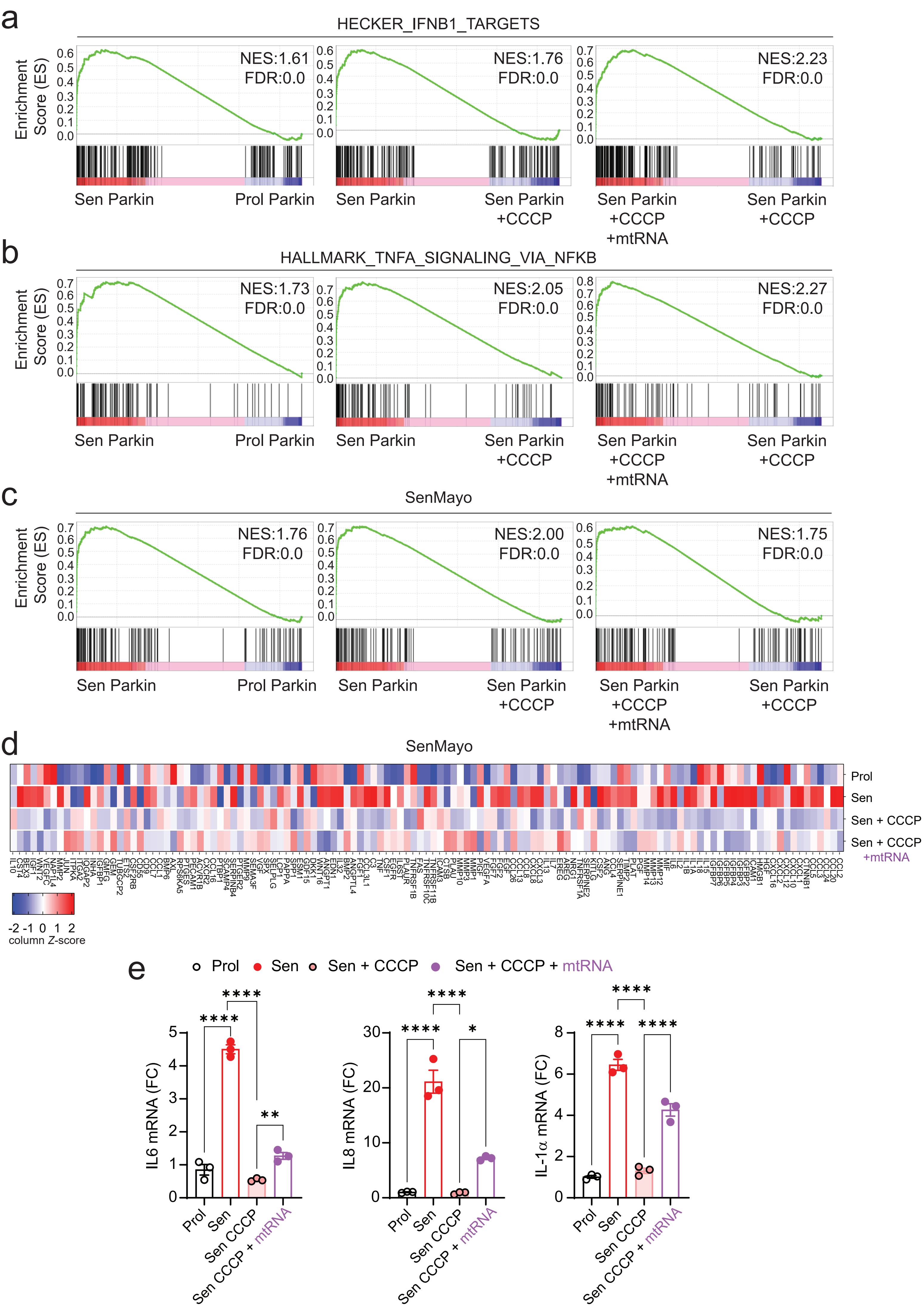
a
HECKER_IFNB1_TARGETS
Enrichment Score (ES)
NES:1.61
FDR:0.0
NES:1.76
FDR:0.0
NES:2.23
FDR:0.0
Sen Parkin
Prol Parkin
Sen Parkin
Sen Parkin +CCCP
Sen Parkin +CCCP +mtRNA
Sen Parkin +CCCP
b
HALLMARK_TNFA_SIGNALING_VIA_NFKB
Enrichment Score (ES)
NES:1.73
FDR:0.0
NES:2.05
FDR:0.0
NES:2.27
FDR:0.0
Sen Parkin
Prol Parkin
Sen Parkin
Sen Parkin +CCCP
Sen Parkin +CCCP +mtRNA
Sen Parkin +CCCP
c
SenMayo
Enrichment Score (ES)
NES:1.76
FDR:0.0
NES:2.00
FDR:0.0
NES:1.75
FDR:0.0
Sen Parkin
Prol Parkin
Sen Parkin
Sen Parkin +CCCP
Sen Parkin +CCCP +mtRNA
Sen Parkin +CCCP
d
SenMayo
Prol
Sen
Sen + CCCP
Sen + CCCP +mtRNA
-2 -1 0 1 2
column Z-score
e
Prol
Sen
Sen + CCCP
Sen + CCCP + mtRNA
IL6 mRNA (FC)
IL8 mRNA (FC)
IL-1α mRNA (FC)
Prol
Sen
Sen CCCP
Sen CCCP + mtRNA

Supplementary Figure 3 - mtRNA reintroduction into mitochondria-depleted senescent cells restores specific inflammatory gene signatures. (a–c) Gene set enrichment analysis (GSEA) showing enrichment of (a) IFNB1 target genes, (b) TNFA–NF-κB signaling genes, and (c) the “SenMayo” SASP signature across indicated comparisons: senescent Parkin-expressing cells (Sen Parkin), mitochondria-depleted senescent cells (Sen Parkin + CCCP), and mitochondria-depleted senescent cells transfected with mtRNA (Sen Parkin + CCCP + mtRNA). NES, normalized enrichment score; FDR, false discovery rate. **(d)** Heatmap of “SenMayo” SASP signature genes from RNA-seq data in proliferating (Prol), senescent (Sen), mitochondria-depleted senescent (Sen + CCCP), and mtRNA-reconstituted mitochondria-depleted senescent cells (Sen + CCCP + mtRNA). Values are column Z-scores. n= 3 (Prol and Sen) and n=6 (Sen + CCCP and Sen + CCCP + mtRNA) independent experiments **(e)** qPCR validation of IL6, IL8, and IL1α expression in the same experimental conditions. Data are mean ± s.e.m., n = 3 independent experiments. Statistical significance was assessed by a one-way ANOVA with Tukey’s post-hoc test. **(e)** (left) $p<0.0001$, $p<0.0001$, $p=0.0098$; (middle) $p<0.0001$, $p<0.0001$, $p=0.0122$; (right) $p<0.0001$, $p<0.0001$, $p<0.0001$.

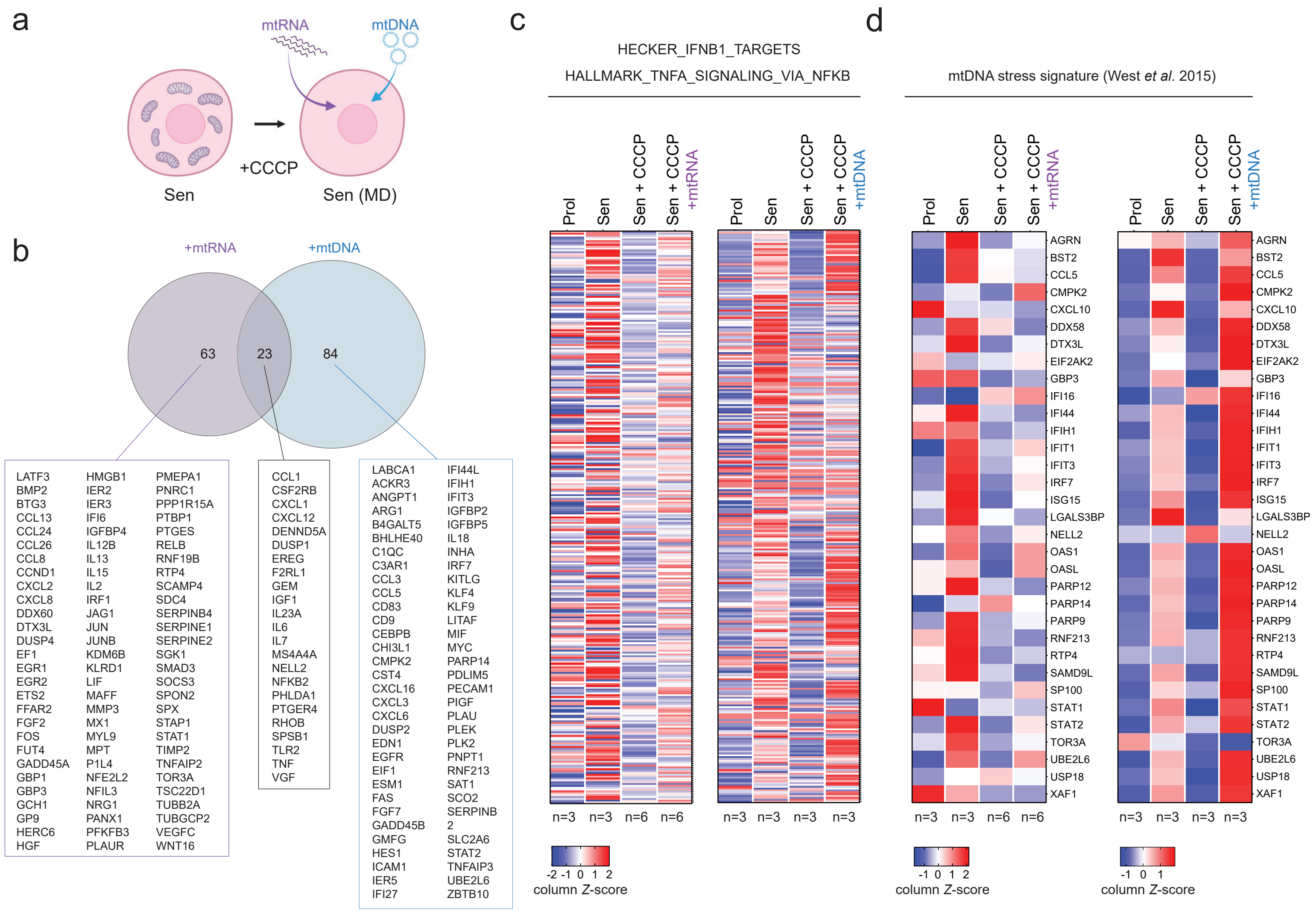
a
mtRNA
mtDNA
Sen
+CCCP
Sen (MD)
b
+mtRNA
+mtDNA
63
23
84
LATF3 BMP2 BTG3 CCL13 CCL24 CCL26 CCL8 CCND1 CXCL2 CXCL8 DDX60 DTX3L DUSP4 EF1 EGR1 EGR2 ETS2 FFAR2 FGF2 FOS FUT4 GADD45A GBP1 GBP3 GCH1 GP9 HERC6 HGF
HMGB1 IER2 IER3 IFI6 IGFBP4 IL12B IL13 IL15 IL2 IRF1 JAG1 JUN JUNB KDM6B KLRD1 LIF MAFF MMP3 MX1 MYL9 MPT P1L4 NFE2L2 NFIL3 NRG1 PANX1 PFKFB3 PLAUR
PMEPA1 PNRC1 PPP1R15A PTBP1 PTGES RELB RNF19B RTP4 SCAMP4 SDC4 SERPINB4 SERPINE1 SERPINE2 SGK1 SMAD3 SOCS3 SPON2 SPX STAP1 STAT1 TIMP2 TNFAIP2 TOR3A TSC22D1 TUBB2A TUBGCP2 VEGFC WNT16
CCL1 CSF2RB CXCL1 CXCL12 DENND5A DUSP1 EREG F2RL1 GEM IGF1 IL23A IL6 IL7 MS4A4A NELL2 NFKB2 PHLDA1 PTGER4 RHOB SPSB1 TLR2 TNF VGF
LABCA1 ACKR3 ANGPT1 ARG1 B4GALT5 BHLHE40 C1QC C3AR1 CCL3 CCL5 CD83 CD9 CEBPB CHI3L1 CMPK2 CST4 CXCL16 CXCL3 CXCL6 DUSP2 EDN1 EGFR EIF1 ESM1 FAS FGF7 GADD45B GMFG HES1 ICAM1 IER5 IFI27
IFI44L IFIH1 IFIT3 IGFBP2 IGFBP5 IL18 INHA IRF7 KITLG KLF4 KLF9 LITAF MIF MYC PARP14 PDLIM5 PECAM1 PIGF PLAU PLEK PLK2 PNPT1 RNF213 SAT1 SCO2 SERPINB 2 SLC2A6 STAT2 TNFAIP3 UBE2L6 ZBTB10
c
HECKER_IFNB1_TARGETS
HALLMARK_TNFA_SIGNALING_VIA_NFKB
Prol
Sen
Sen + CCCP
Sen + CCCP +mtRNA
Sen + CCCP +mtDNA
n=3 n=3 n=6 n=6
n=3 n=3 n=3 n=3
-2 -1 0 1 2
column Z-score
d
mtDNA stress signature (West et al. 2015)
AGRN BST2 CCL5 CMPK2 CXCL10 DDX58 DTX3L EIF2AK2 GBP3 IFI16 IFI44 IFIH1 IFIT1 IFIT3 IRF7 ISG15 LGALS3BP NELL2 OAS1 OASL PARP12 PARP14 PARP9 RNF213 RTP4 SAMD9L SP100 STAT1 STAT2 TOR3A UBE2L6 USP18 XAF1
n=3 n=3 n=6 n=6
n=3 n=3 n=3 n=3
-1 0 1 2
-1 0 1
column Z-score

Supplementary Figure 4 - mtRNA and mtDNA activate overlapping and distinct inflammatory programs in mitochondria-depleted senescent cells. (a) Schematic showing experimental design: senescent cells (Sen) were depleted of mitochondria by Parkin-mediated mitophagy using CCCP (Sen + CCCP; MD, mitochondria-depleted), followed by transfection with isolated mtRNA or mtDNA. Created in BioRender. Victorelli, S. (2025) https://BioRender.com/xa8sjtz. **(b)** Venn diagram showing overlap between inflammation-related genes induced by mtRNA (purple) or mtDNA (blue) in mitochondria-depleted senescent cells. After DESeq analysis with DESeq2 (v 1.46.0), a threshold of padj <0.05 and FC>0 was used as input, while the gene sets HALLMARK_TNFA_SIGNALING_VIA_NFKB (M5890), HECKER_IFNB1_TARGETS (M3010), SenMayo (PMID: 35974106) and West *et a*l. (PMID: 25642965) were filtered. Lists of genes uniquely or commonly induced are shown below. **(c)** Heatmaps of RNA-seq data showing expression of IFNB1 target genes (HECKER_IFNB1_TARGETS) and TNFA–NF-κB signaling pathway genes (HALLMARK_TNFA_SIGNALING_VIA_NFKB) in proliferating (Prol), senescent (Sen), mitochondria-depleted senescent (Sen + CCCP), and nucleic acid-reconstituted cells (Sen + CCCP + mtRNA or Sen + CCCP + mtDNA). Values are column Z-scores. **(d)** Heatmaps of the previously defined "mtDNA stress" signature (West *et al*., 2015) in the same conditions as in (c), showing that mtDNA reintroduction restores and amplifies the signature, whereas mtRNA has minimal effect. Values are column Z-scores. **(a-d)** (for mtRNA dataset) n=3 (Prol and Sen) and n=6 (Sen + CCCP and Sen + CCCP + mtRNA); (for mtDNA dataset) n=3 independent experiments.

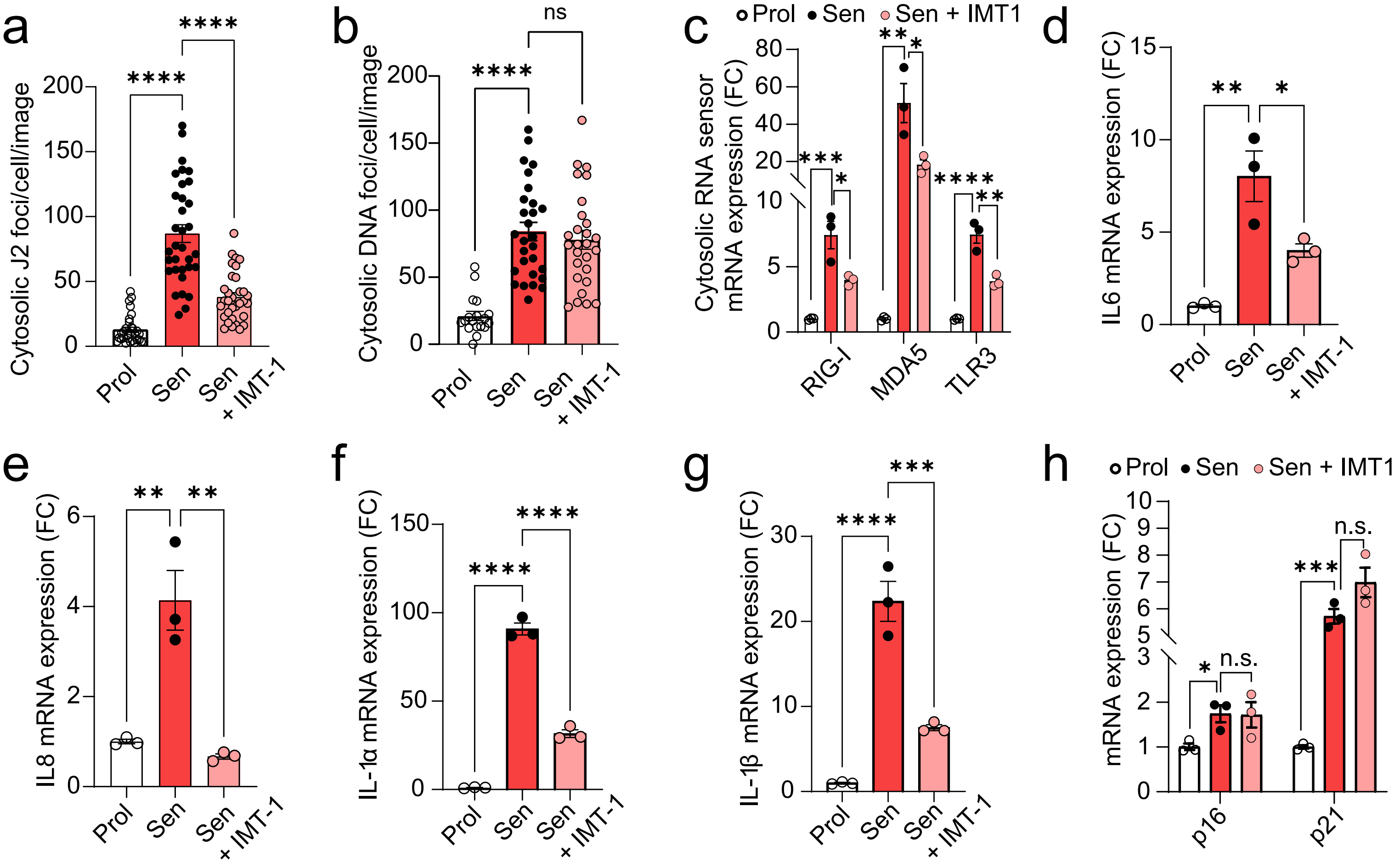

a
b
c
d
e
f
g
h
Cytosolic J2 foci/cell/image
Cytosolic DNA foci/cell/image
Cytosolic RNA sensor mRNA expression (FC)
IL6 mRNA expression (FC)
IL8 mRNA expression (FC)
IL-1α mRNA expression (FC)
IL-1β mRNA expression (FC)
mRNA expression (FC)
Prol
Sen
Sen + IMT-1
Sen + IMT1
RIG-I
MDA5
TLR3
p16
p21
ns
n.s.

Supplementary Figure 5 - Pharmacological inhibition of POLRMT decreases cytosolic leakage of mtRNA and the SASP during senescence. (a) The number of cytosolic dsRNA foci (J2 antibody) and **(b)** cytosolic DNA (anti-DNA antibody) in proliferating (Prol) and senescent (Sen) cells with and without IMT-1 treatment. Data are mean ± s.e.m. (a). n = 30 (Prol), 33 (Sen) and 32 (Sen + IMT-1) cells; (b) n=18 (Prol), n=28 (Sen) and n=27 cell (Sen + IMT-1) analyzed over 3 independent experiments. **(c)** cytosolic RNA sensor expression in proliferating and senescent cells with or without POLRMT inhibition. mRNA expression of SASP factors **(d)** IL6, **(e)** IL8, **(f)** IL-1α, **(g)** IL-1β, and the cyclin-dependent kinase inhibitors **(h)** p16 and p21 in proliferating and senescent cells with and without IMT-1 treatment. (c-h) n=3 independent experiments. Data are mean ± s.e.m. Statistical significance was assessed using a one-way ANOVA followed by Tukey's multiple comparison test. **(a)** $p<0.0001$, $p<0.0001$; **(b)** $p<0.0001$, $p=0.9265$; **(c)** $p=0.0008$, $p=0.0183$, $p=0.003$, $p=0.022$, $p<0.0001$, $p=0.002$; **(d)** $p=0.0022$, $p=0.0308$; **(e)** $p=0.0029$, $p=0.0017$; **(f)** $p<0.0001$, $p<0.0001$; **(g)** $p<0.0001$, $p=0.0006$; **(h)** $p=0.0217$, $p=0.9953$, $p=0.0002$, $p=0.0982$.

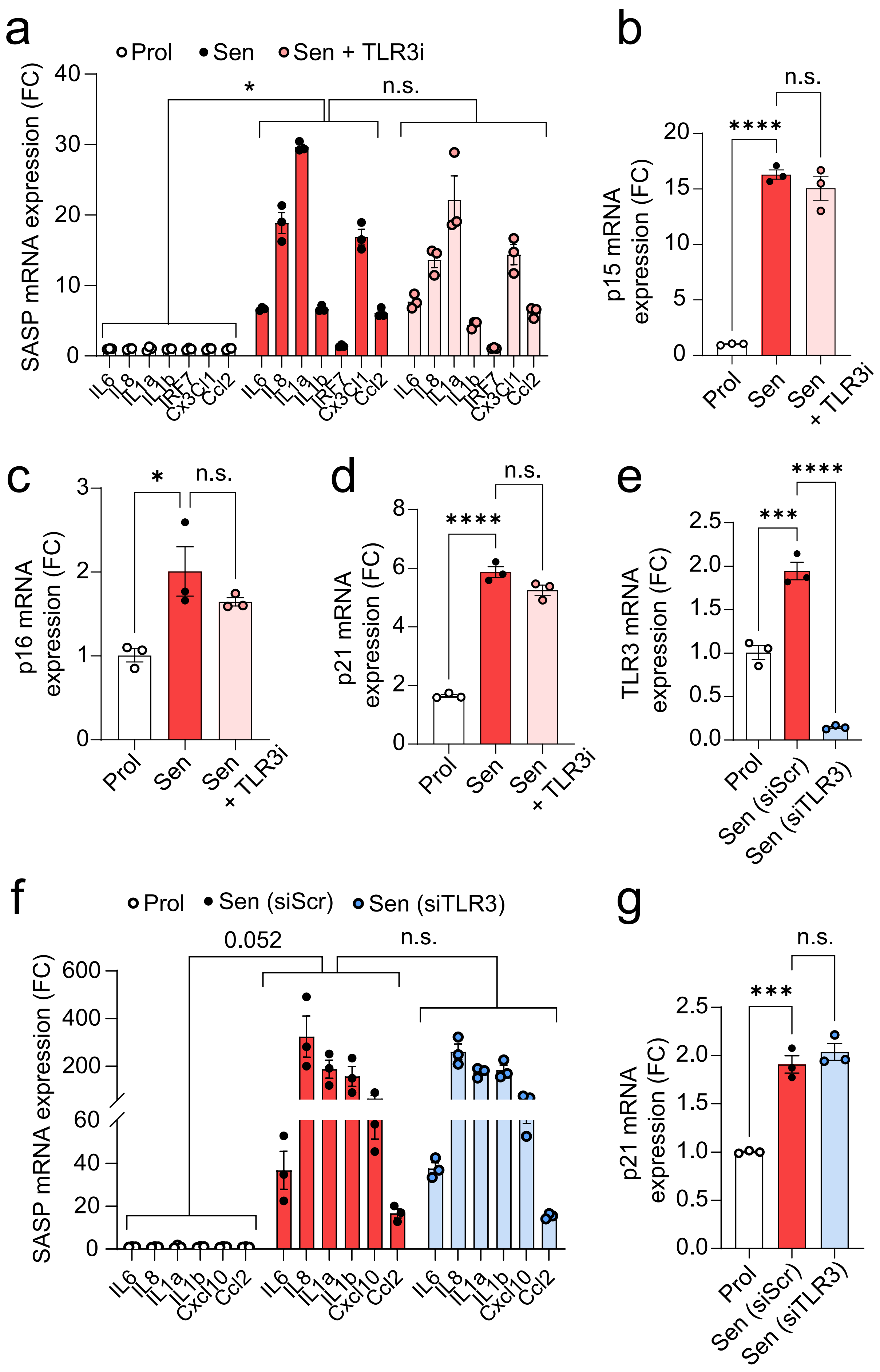
a
Prol
Sen
Sen + TLR3i
SASP mRNA expression (FC)
*
n.s.
IL6
IL8
IL1a
IL1b
IRF7
Cx3Cl1
Ccl2
b
p15 mRNA expression (FC)
****
n.s.
Prol
Sen
Sen + TLR3i
c
p16 mRNA expression (FC)
*
n.s.
d
p21 mRNA expression (FC)
****
n.s.
e
TLR3 mRNA expression (FC)
***
****
Prol
Sen (siScr)
Sen (siTLR3)
f
Prol
Sen (siScr)
Sen (siTLR3)
SASP mRNA expression (FC)
0.052
n.s.
IL6
IL8
IL1a
IL1b
Cxcl10
Ccl2
g
p21 mRNA expression (FC)
***
n.s.

Supplementary Figure 6 - TLR3 inhibition or knockdown does not significantly reduce SASP expression in senescent cells. mRNA expression of **(a)** the indicated SASP factors, **(b)** p15, **(c)** p16 and **(d)** p21 in proliferating and senescent cells with and without treatment with TLR3/dsRNA complex inhibitor (TLR3i). **(e)** qPCR analysis confirming TLR3 knockdown in senescent cells transfected with siRNA targeting TLR3 (siTLR3) compared to scrambled control (siScr). **(f)** qPCR analysis of indicated SASP mRNA levels in proliferating, siScr-treated senescent cells, and siTLR3-treated senescent cells. **(g)** qPCR analysis of p21 mRNA levels in the same samples as in (f). (a-g) Data are mean ± s.e.m., n = 3 independent experiments. Statistical significance was assessed using Nested one-way ANOVA (a, f) and one-way ANOVA (b-e, g) followed by Tukey's multiple comparison test. **(a)** $p=0.0198$, $p=0.8062$; **(b)** $p<0.0001$, $p=0.4439$; **(c)** $p=0.0175$, $p=0.3819$; **(d)** $p<0.0001$, $p=0.0599$; **(e)** $p=0.0003$, $p<0.0001$; **(f)** $p=0.0523$, $p=0.9847$; **(g)** $p=0.0003$, $p=0.4610$.

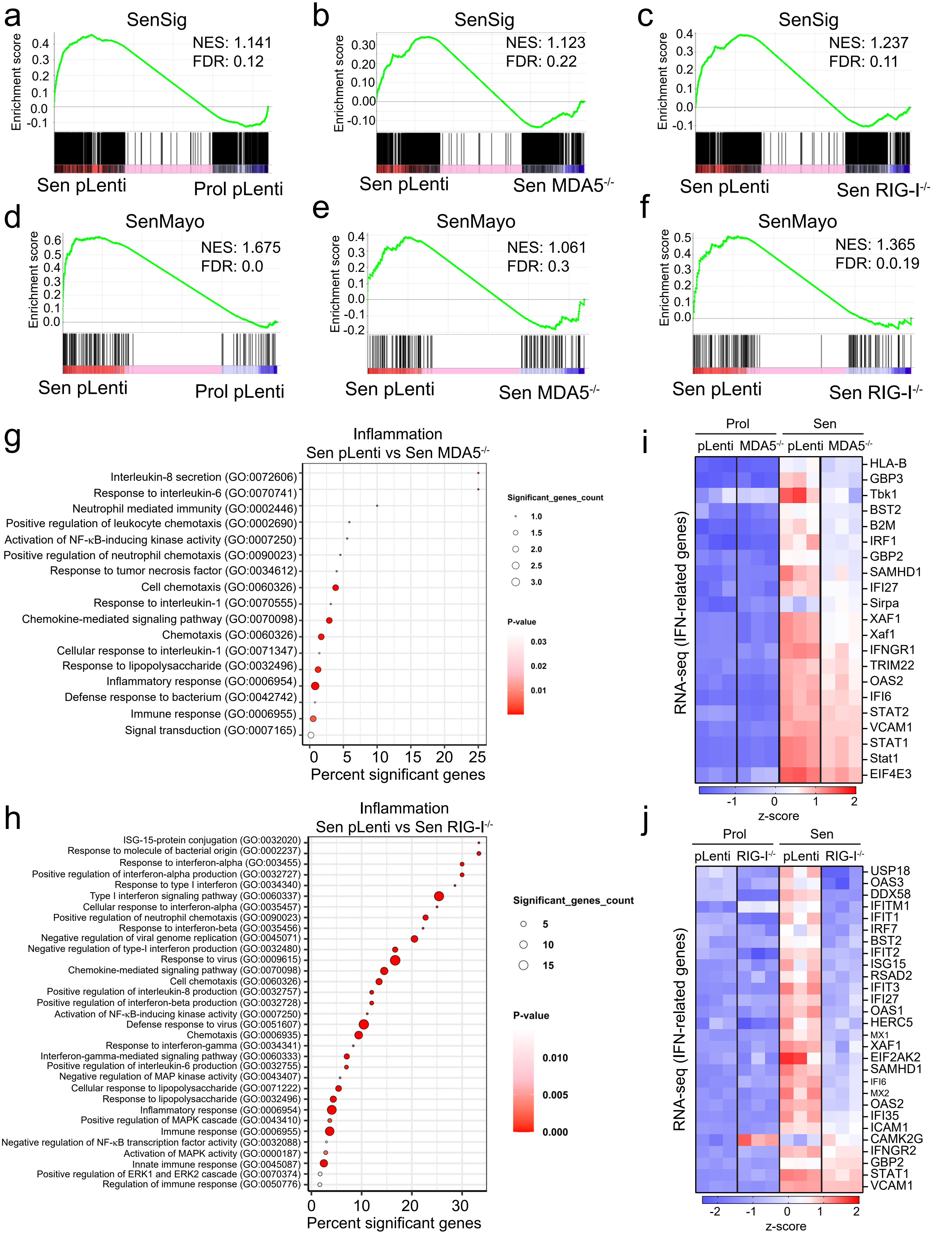

a
SenSig
NES: 1.141
FDR: 0.12
Enrichment score
Sen pLenti
Prol pLenti
b
SenSig
NES: 1.123
FDR: 0.22
Sen pLenti
Sen MDA5-/-
c
SenSig
NES: 1.237
FDR: 0.11
Sen pLenti
Sen RIG-I-/-
d
SenMayo
NES: 1.675
FDR: 0.0
Sen pLenti
Prol pLenti
e
SenMayo
NES: 1.061
FDR: 0.3
Sen pLenti
Sen MDA5-/-
f
SenMayo
NES: 1.365
FDR: 0.0.19
Sen pLenti
Sen RIG-I-/-
g
Inflammation
Sen pLenti vs Sen MDA5-/-
Interleukin-8 secretion (GO:0072606)
Response to interleukin-6 (GO:0070741)
Neutrophil mediated immunity (GO:0002446)
Positive regulation of leukocyte chemotaxis (GO:0002690)
Activation of NF-κB-inducing kinase activity (GO:0007250)
Positive regulation of neutrophil chemotaxis (GO:0090023)
Response to tumor necrosis factor (GO:0034612)
Cell chemotaxis (GO:0060326)
Response to interleukin-1 (GO:0070555)
Chemokine-mediated signaling pathway (GO:0070098)
Chemotaxis (GO:0060326)
Cellular response to interleukin-1 (GO:0071347)
Response to lipopolysaccharide (GO:0032496)
Inflammatory response (GO:0006954)
Defense response to bacterium (GO:0042742)
Immune response (GO:0006955)
Signal transduction (GO:0007165)
Percent significant genes
Significant_genes_count
P-value
h
Inflammation
Sen pLenti vs Sen RIG-I-/-
ISG-15-protein conjugation (GO:0032020)
Response to molecule of bacterial origin (GO:0002237)
Response to interferon-alpha (GO:003455)
Positive regulation of interferon-alpha production (GO:0032727)
Response to type I interferon (GO:0034340)
Type I interferon signaling pathway (GO:0060337)
Cellular response to interferon-alpha (GO:0035457)
Positive regulation of neutrophil chemotaxis (GO:0090023)
Response to interferon-beta (GO:0035456)
Negative regulation of viral genome replication (GO:0045071)
Negative regulation of type-I interferon production (GO:0032480)
Response to virus (GO:0009615)
Chemokine-mediated signaling pathway (GO:0070098)
Cell chemotaxis (GO:0060326)
Positive regulation of interleukin-8 production (GO:0032757)
Positive regulation of interferon-beta production (GO:0032728)
Activation of NF-κB-inducing kinase activity (GO:0007250)
Defense response to virus (GO:0051607)
Chemotaxis (GO:0006935)
Response to interferon-gamma (GO:0034341)
Interferon-gamma-mediated signaling pathway (GO:0060333)
Positive regulation of interleukin-6 production (GO:0032755)
Negative regulation of MAP kinase activity (GO:0043407)
Cellular response to lipopolysaccharide (GO:0071222)
Response to lipopolysaccharide (GO:0032496)
Inflammatory response (GO:0006954)
Positive regulation of MAPK cascade (GO:0043410)
Immune response (GO:0006955)
Negative regulation of NF-κB transcription factor activity (GO:0032088)
Activation of MAPK activity (GO:0000187)
Innate immune response (GO:0045087)
Positive regulation of ERK1 and ERK2 cascade (GO:0070374)
Regulation of immune response (GO:0050776)
Percent significant genes
Significant_genes_count
P-value
i
Prol
Sen
pLenti
MDA5-/-
RNA-seq (IFN-related genes)
HLA-B
GBP3
Tbk1
BST2
B2M
IRF1
GBP2
SAMHD1
IFI27
Sirpa
XAF1
Xaf1
IFNGR1
TRIM22
OAS2
IFI6
STAT2
VCAM1
STAT1
Stat1
EIF4E3
z-score
j
Prol
Sen
pLenti
RIG-I-/-
RNA-seq (IFN-related genes)
USP18
OAS3
DDX58
IFITM1
IFIT1
IRF7
BST2
IFIT2
ISG15
RSAD2
IFIT3
IFI27
OAS1
HERC5
MX1
XAF1
EIF2AK2
SAMHD1
IFI6
MX2
OAS2
IFI35
ICAM1
CAMK2G
IFNGR2
GBP2
STAT1
VCAM1
z-score

Supplementary Figure 7 - Deletion of MDA5 and RIG-I in senescent cells decreases SenMayo and SenSig gene signatures and a subset of interferon-associated genes. (a–c) Gene set enrichment analysis (GSEA) of the "SenSig" SASP signature in proliferating (Prol) and senescent (Sen) cells, and in senescent cells following knockout of MDA5 ($MDA5^{-/-}$) or RIG-I ($RIG\text{-}I^{-/-}$). NES, normalized enrichment score; FDR, false discovery rate. **(d–f)** GSEA of the "SenMayo" SASP signature in the same conditions as in (a–c). **(g,h)** Gene ontology (GO) term enrichment analysis of inflammation-related pathways significantly downregulated in senescent cells after MDA5 loss (g) or RIG-I loss (h). **(i,j)** Column clustered heatmaps of RNA-seq data showing expression of interferon-related genes in proliferating and senescent cells after MDA5 loss (i) or RIG-I loss (j). Values are row Z-scores. **(a-j)** n=3 independent experiments.

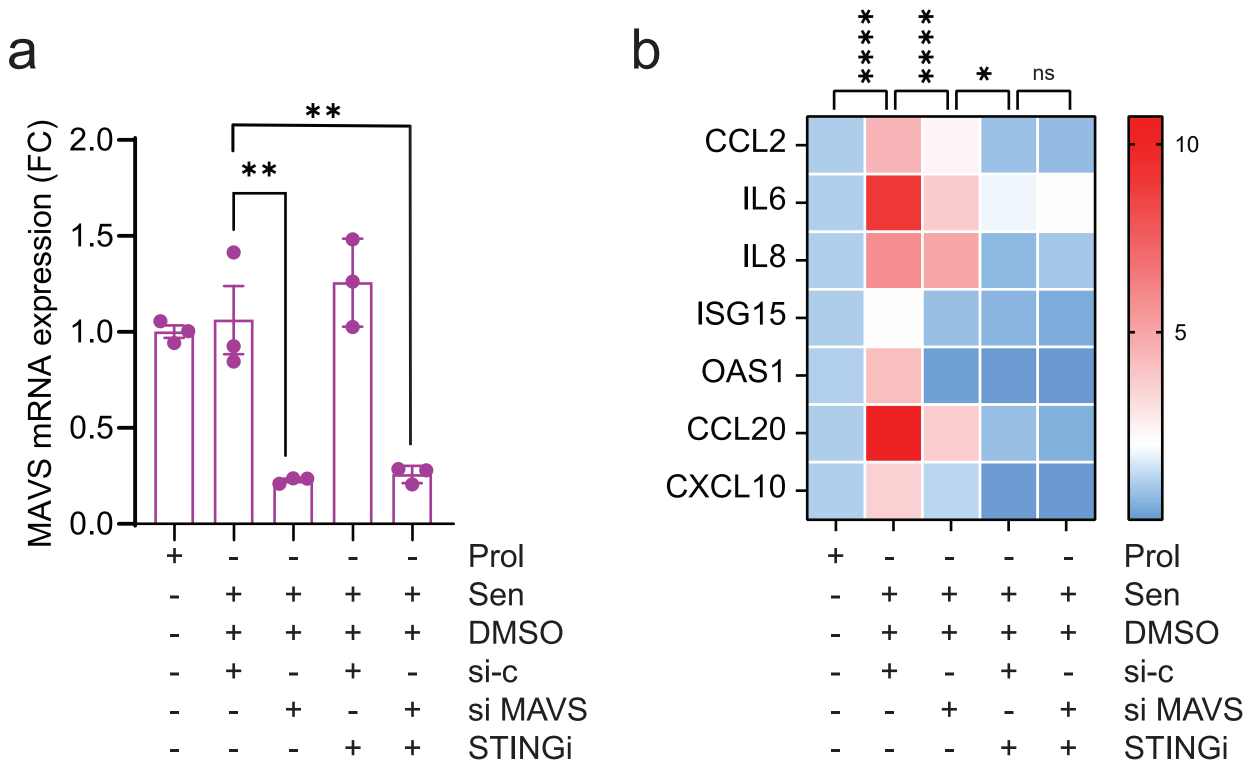
a
MAVS mRNA expression (FC)
2.0
1.5
1.0
0.5
0.0
**
**
Prol
Sen
DMSO
si-c
si MAVS
STINGi
b
****
****
*
ns
CCL2
IL6
IL8
ISG15
OAS1
CCL20
CXCL10
10
5
Prol
Sen
DMSO
si-c
si MAVS
STINGi

Supplementary Figure 8 - MAVS knockdown and STING inhibition are not additive in reducing SASP expression. (a) qPCR analysis of MAVS mRNA levels in proliferating (Prol) and senescent (Sen) cells treated with vehicle (DMSO), scrambled siRNA control (si-c), siRNA targeting MAVS (siMAVS), and/or the STING inhibitor SN-011 (STINGi). $^{**}P < 0.01$, $^{***}P < 0.001$. one-way ANOVA with Tukey's post-hoc test. **(b)** Heatmap showing expression (fold change) of selected SASP and interferon-stimulated genes (CCL2, IL6, IL8, ISG15, OAS1, CCL20, CXCL10) in the same experimental conditions. (a, b) Data are mean ± s.e.m., n = 3 independent experiments. Statistical significance was assessed by **(a)** one-way ANOVA and **(b)** nested one-way ANOVA followed by Tukey's multi-comparison test. **(a)** p=0.0012, p=0.0015; **(b)** p<0.0001, p<0.0001, p=0.0104, p>0.9999.

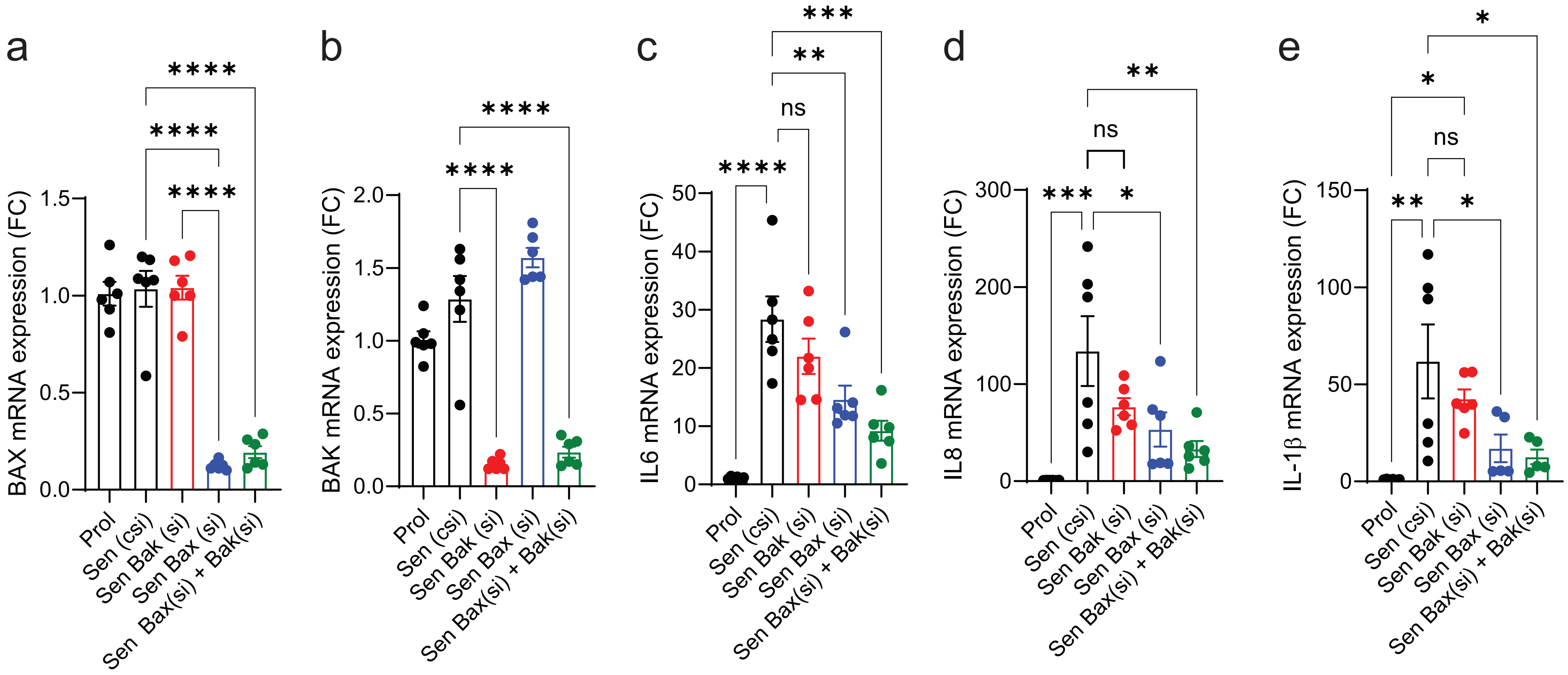

a
BAX mRNA expression (FC)
0.0
0.5
1.0
1.5
****
****
****
Prol
Sen (csi)
Sen Bak (si)
Sen Bax (si)
Sen Bax(si) + Bak(si)
b
BAK mRNA expression (FC)
0.0
0.5
1.0
1.5
2.0
****
****
Prol
Sen (csi)
Sen Bak (si)
Sen Bax (si)
Sen Bax(si) + Bak(si)
c
IL6 mRNA expression (FC)
0
10
20
30
40
50
***
**
ns
****
Prol
Sen (csi)
Sen Bak (si)
Sen Bax (si)
Sen Bax(si) + Bak(si)
d
IL8 mRNA expression (FC)
0
100
200
300
**
ns
***
*
Prol
Sen (csi)
Sen Bak (si)
Sen Bax (si)
Sen Bax(si) + Bak(si)
e
IL-1β mRNA expression (FC)
0
50
100
150
*
*
ns
**
*
Prol
Sen (csi)
Sen Bak (si)
Sen Bax (si)
Sen Bax(si) + Bak(si)

Supplementary Figure 9 - BAX, but not BAK, is required for SASP expression in senescent cells. (a,b) qPCR analysis confirming knockdown efficiency of BAX (a) and BAK (b) mRNA in proliferating (Prol) and senescent (Sen) cells transfected with scrambled siRNA (csi) or siRNAs targeting BAK (Bak(si)), BAX (Bax(si)), or both BAX and BAK (Bax(si) + Bak(si)). **(c–e)** qPCR analysis of mRNA expression for IL6 (c), IL8 (d), and IL1β (e) in the same conditions as in (a,b). Knockdown of BAX alone significantly reduced SASP factor expression, whereas BAK knockdown had little effect. (a-e) Data are mean ± s.e.m., n = 6 independent experiments (a-d); n=6 (Prol, Sen (csi), Sen Bak (si), n=5 (Sen Bax (si) and Bax(si) + Bak (si)) (e). One-way ANOVA with Tukey's post-hoc test. **(a)** $p<0.0001$, $p<0.0001$, $p<0.0001$; **(b)** $p<0.0001$, $p<0.0001$; **(c)** $p<0.0001$, $p=0.4245$, $p=0.0069$, $p=0.0002$; **(d)** $p=0.0003$, $p=0.2230$, $p=0.0383$, $p=0.0064$; **(e)** $p=0.0016$, $p=0.0424$, $p=0.6264$, $p=0.0349$, $p=0.0176$.

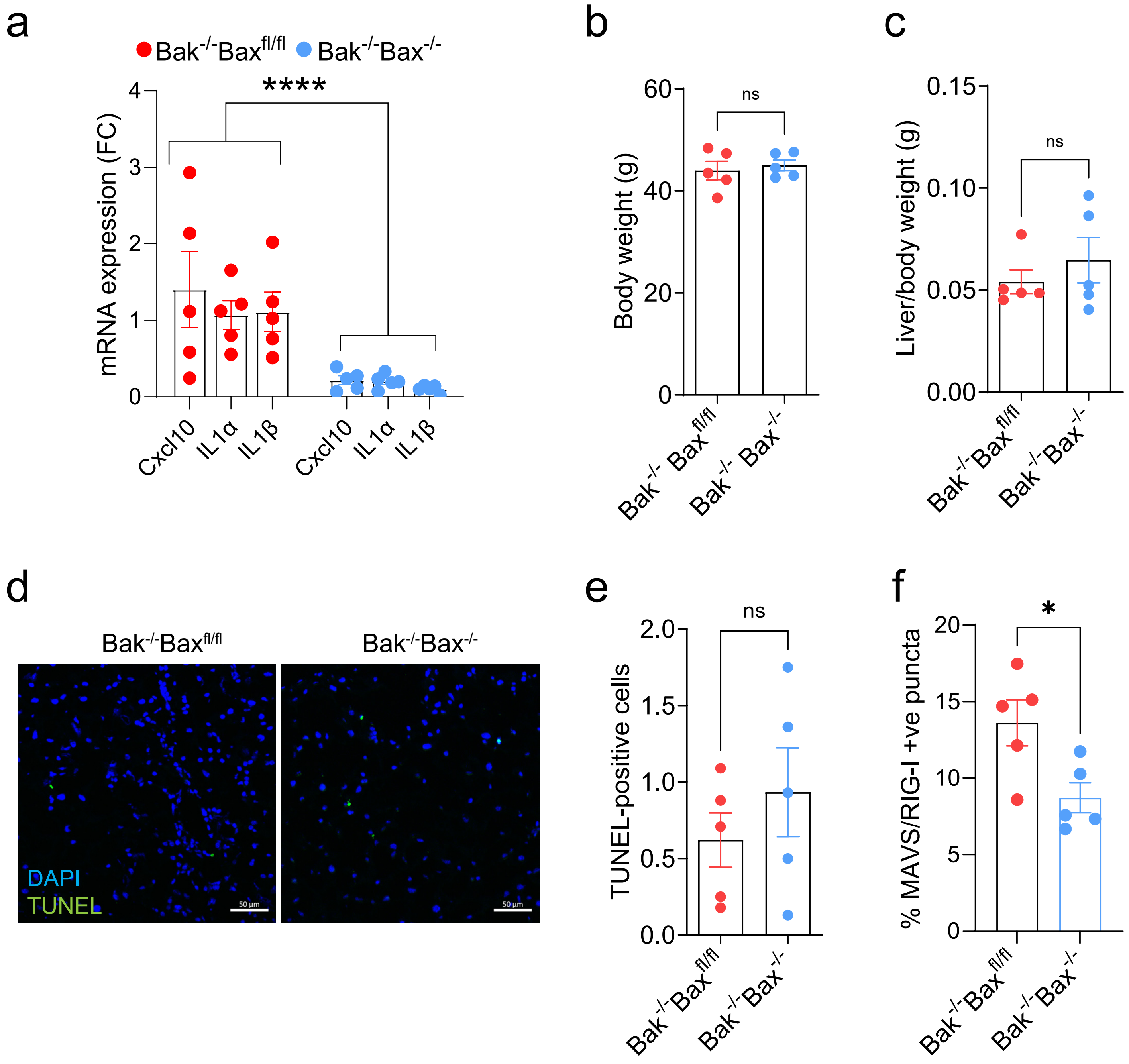

a
Bak-/-Baxfl/fl
Bak-/-Bax-/-
****
mRNA expression (FC)
0
1
2
3
4
Cxcl10
IL1α
IL1β
Cxcl10
IL1α
IL1β
b
ns
Body weight (g)
0
20
40
60
Bak-/- Baxfl/fl
Bak-/- Bax-/-
c
ns
Liver/body weight (g)
0.00
0.05
0.10
0.15
Bak-/-Baxfl/fl
Bak-/-Bax-/-
d
Bak-/-Baxfl/fl
Bak-/-Bax-/-
DAPI
TUNEL
50 µm
50 µm
e
ns
TUNEL-positive cells
0.0
0.5
1.0
1.5
2.0
Bak-/-Baxfl/fl
Bak-/-Bax-/-
f
*
% MAVS/RIG-I +ve puncta
0
5
10
15
20
Bak-/-Baxfl/fl
Bak-/-Bax-/-

Supplementary Figure 10 – Hepatocyte-specific BAX/BAK$^{-/-}$ does not affect body weight and markers of apoptosis in the liver during MASH. (a) qPCR quantification of SASP-related factors in livers from FFC-fed Bak$^{-/-}$ Bax$^{fl/fl}$ and Bak$^{-/-}$ Bax$^{-/-}$ mice. **(b)** Body weight and **(c)** liver/body weight ratio of FFC-fed Bak$^{-/-}$ Bax$^{fl/fl}$ and Bak$^{-/-}$ Bax$^{-/-}$ mice. **(d)** Representative images of TUNEL staining (green) with DAPI (blue) in liver sections from Bak$^{-/-}$ Bax$^{fl/fl}$ and Bax$^{-/-}$ Bak$^{-/-}$ mice MASH mice. Scale bar = 50μm. **(e)** Number of TUNEL-positive cells per field in the liver of Bak$^{-/-}$ Bax$^{fl/fl}$ and Bax$^{-/-}$ Bak$^{-/-}$ mice MASH mice. n=5 mice per group. **(f)** Quantification of the percentage of MAVS colocalizing with RIG-I in liver tissue using Proximity Ligation Assay (PLA). (a-f) Data are mean ± s.e.m. ; n=5 mice *per* group. Statistical significance was assessed by a Nested t-test (a) and two-sided Student's unpaired t-tests (b,c,e,f). **(a)** p<0.0001; **(b)** p=0.6427; **(c)** p=0.4220; **(e)** p=0.3854; **(f)** p=0.0261.

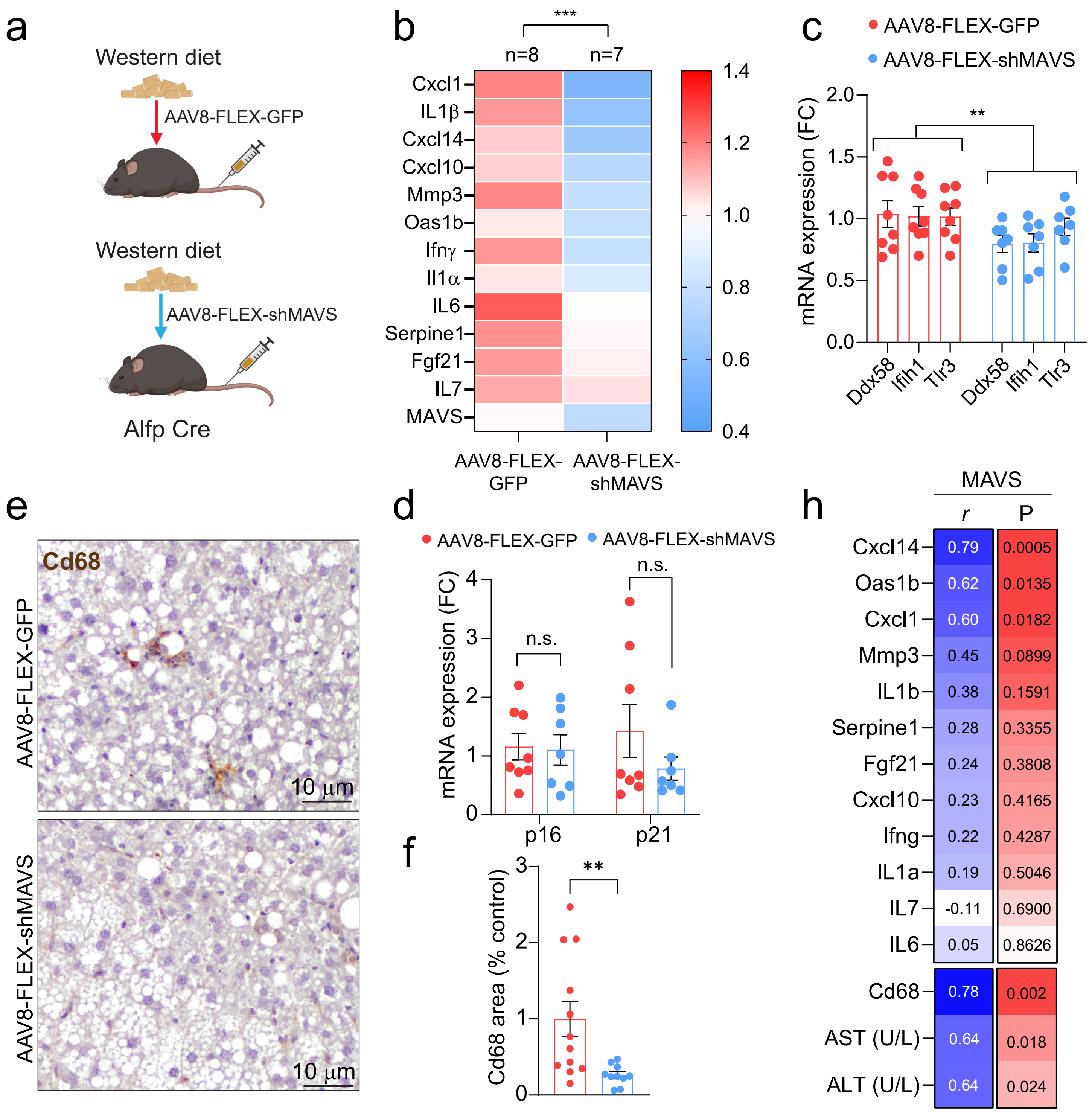

a
Western diet
AAV8-FLEX-GFP
Western diet
AAV8-FLEX-shMAVS
Alfp Cre
b
***
n=8
n=7
Cxcl1
IL1β
Cxcl14
Cxcl10
Mmp3
Oas1b
Ifnγ
Il1α
IL6
Serpine1
Fgf21
IL7
MAVS
AAV8-FLEX-GFP
AAV8-FLEX-shMAVS
1.4
1.2
1.0
0.8
0.6
0.4
c
AAV8-FLEX-GFP
AAV8-FLEX-shMAVS
mRNA expression (FC)
**
Ddx58
Ifih1
Tlr3
Ddx58
Ifih1
Tlr3
e
Cd68
AAV8-FLEX-GFP
AAV8-FLEX-shMAVS
10 μm
10 μm
d
AAV8-FLEX-GFP
AAV8-FLEX-shMAVS
mRNA expression (FC)
n.s.
n.s.
p16
p21
f
Cd68 area (% control)
**
h
MAVS
r
P
Cxcl14 0.79 0.0005
Oas1b 0.62 0.0135
Cxcl1 0.60 0.0182
Mmp3 0.45 0.0899
IL1b 0.38 0.1591
Serpine1 0.28 0.3355
Fgf21 0.24 0.3808
Cxcl10 0.23 0.4165
Ifng 0.22 0.4287
IL1a 0.19 0.5046
IL7 -0.11 0.6900
IL6 0.05 0.8626
Cd68 0.78 0.002
AST (U/L) 0.64 0.018
ALT (U/L) 0.64 0.024

Supplementary Figure 11 - Hepatocyte-specific MAVS knockdown reduces inflammatory gene expression and macrophage infiltration in FFC-fed mice. (a) Schematic of experimental design. Mice expressing hepatocyte-specific Cre recombinase (Alfp-Cre) were fed an FFC diet and injected with AAV8-FLEX-GFP (control) or AAV8-FLEX-shMAVS to knock down MAVS in hepatocytes. Created in BioRender. Victorelli, S. (2025) https://BioRender.com/u4gyogf **(b)** Heatmap showing fold-change expression of inflammatory and SASP-associated genes in livers from AAV8-FLEX-GFP (n=8) and AAV8-FLEX-shMAVS (n=7) mice, determined by qPCR. Data are scaled by row; red indicates higher and blue lower expression (Nested two-tailed t-test). **(c)** Relative mRNA expression (fold change, FC) of RNA sensors *Ddx58*, *Ifih1*, and *Tlr3* showing significant reductions in shMAVS mice (Nested two-tailed t-test). **(d)** mRNA expression of senescence-associated cell cycle inhibitors *p16* and *p21*, showing no significant changes upon MAVS knockdown (two-tailed unpaired t-test). **(e)** Representative liver sections immunostained for Cd68 in GFP control and shMAVS mice. Scale bar, 10 μm. **(f)** Quantification of Cd68-positive area as percentage of control, showing reduced macrophage infiltration in shMAVS livers (two-tailed unpaired t-test). **(h)** Pearson correlation coefficients (r) and *P*-values between MAVS expression and indicated inflammatory genes, Cd68, and serum transaminases (AST, ALT). Significant correlations are indicated in red.. Data are mean ± s.e.m. (c, d, h) n=8 GFP and n=7 shMAVS mice; (f) n=12 GFP and n=10 shMAVS mice. **(b)** p=0.0001; **(c)** p=0.0074; **(d)** p=0.8776, p=0.2353; **(f)** p=0.01.

qPCR Primers		
Name	Source	Product number
HUMAN		
CCL2	IDT	Hs.PT.58.45467977
IFNB1	IDT	Hs.PT.58.39481063.g
IL6	IDT	Hs.PT.58.40226675
IL8	IDT	Hs.PT.58.39926886.g
IL-1a	IDT	Hs.PT.58.40913627
IL-1b	IDT	Hs.PT.58.1518186
Cx3Cl1	IDT	Hs.PT.58.19601997
IRF7	IDT	Hs.PT.58.39380279.g
DDX58 (RIG-1)	IDT	Hs.PT.58.4273674
IFIH1 (MDA5)	IDT	Hs.PT.58.1224165
TLR3	IDT	Hs.PT.58.25887499.g
MAVS	IDT	Hs.PT.58.21315930
p15 (CDKN2B)	IDT	Hs.PT.58.4919581
p16 (CDKN2A)	IDT	Hs.PT.58.24382464.g
p21 (CDKN1A)	IDT	Hs.PT.58.38492863.g
ISG-15	IDT	Hs.PT.58.39185901.g
MT-COI	Thermo Scientific	Hs02596864_g1
MT-CYB	Thermo Scientific	Hs02596867_g1
MT-ND5	Thermo Scientific	Hs02596878_g1
MT-ND6	Thermo Scientific	Hs02596879_g1
Bax	IDT	Hs.PT.56a.19141193.g
Bak1	IDT	Hs.PT.56a.40435467
Cxcl10	IDT	Hs.PT.58.3790956.g
TBP	IDT	Hs.PT.39a.22214825
MOUSE		
Ccl2	IDT	Mm.PT.58.42151692
CXCL10	IDT	Mm.PT.58.43575827
IL-1a	IDT	Mm.PT.58.32778767
IL-1b	IDT	Mm.PT.58.41616450
OAS1b	IDT	Mm.PT.56a.10289138.g
OASl2	IDT	Mm.PT.56a.17167264
Cd45	IDT	Mm.PT.58.7583849
Cd68	IDT	Mm.PT.58.32698807
DDX58 (RIG-I)	IDT	Mm.PT.58.9774198
IFIH1 (MDA5)	IDT	Mm.PT.58.13626888
TLR3	IDT	Mm.PT.58.8085919
Bax	IDT	Mm.PT.58.14012210
Cxcl1	IDT	Mm.PT.58.42076891
Cxcl14	IDT	Mm.PT.58.21980826
Mmp3	IDT	Mm.PT.58.9719290
Ifng	IDT	Mm.PT.58.41769240
IL6	IDT	Mm.PT.58.10005566

Serpine1	IDT	Mm.PT.58.6413525
Fgf21	IDT	Mm.PT.58.29365871.g
IL7	IDT	Mm.PT.58.10325839
MAVS	IDT	Mm.PT.58.32692265
p16 (Cdkn2a)	IDT	Mm.PT.58.42804808
p21 (Cdkn1a)	IDT	Mm.PT.58.5884610
HPRT	IDT	Mm.PT.39a.22214828
**SYBR Green Primers**		
TIMP1	IDT	Forward: AGGTGGTCTCGTTGATTTCT<br>Reverse: GTAAGGCCTGTAGCTGTGCC
Collagen1a1	IDT	Forward: GCTCCTCTTAGGGGCCACT<br>Reverse: CCACGTCTCACCATTGGGG
aSMA	IDT	Forward: GTCCCAGACATCAGGGAGTAA<br>Reverse: TCGGATACTTCAGCGTCAGGA

Supplementary Table 1 – List of qPCR primers used.

Western Blot Antibodies			
**Primary antibodies**			
**Name**	**Source**	**Product number**	**Dilution**
MAVS	Santa Cruz	SC166583	1:1000
p21	Abcam	Ab109199	1:1000
p16	Cell Signalling	92803S	1:1000
MDA5 (IFIH1)	Abcam	Ab126630	1:1000
RIG-1 (DDX58)	Abcam	Ab180675	1:1000
TLR3	Abcam	Ab62566	1:1000
Actin	Sigma	A2066	1:1000
TOM20	Sigma	HPA011562	1:1000
GAPDH	Cell Signalling	5174S	1:1000
PCNA	Abcam	Ab29	1:1000
Tubulin	Cell Signalling	2146S	1:1000
BAX	Cell Signalling	2772T	1:1000
BAK	Cell Signalling	12105S	1:1000
HSP90	Cell Signalling	4874	1:1000
NDUFB8	Abcam	Ab110242	1:1000
UQCRC2	Abcam	Ab14745	1:1000
COX IV	Abcam	ab33985	1:1000
**Secondary antibodies**			
Goat Anti-Rabbit HRP Conjugated	Sigma Aldrich	A0545	1:5000
Goat Anti-Mouse HRP Conjugated	Sigma Aldrich	A2554	1:5000

Supplementary Table 2 – List of antibodies used for Western blot.

Immunocytochemistry and Immunohistochemistry Antibodies			
**Primary antibodies**			
**Name**	**Source**	**Product number**	**Dilution**
Anti-MAVS mouse monoclonal antibody	Santa Cruz	Sc-166583	1:100
Anti-dsRNA J2 mouse monoclonal antibody	Jena Bioscience	RNT-SCI-10010200	1:1000
Anti-TOM20 rabbit polyclonal antibody	Millipore Sigma	HPA011562	1:200
Anti-DNA mouse monoclonal antibody	Millipore Sigma	CBL186	1:100
Anti-Phosphorylated Histone H2A.X (S139) (20E3)	Cell Signaling	9718S	1:400
Anti-MAVS (E-3) mouse monoclonal antibody	Santa Cruz	Sc-166583	1:100
Anti-RIG-1/DDX58 rabbit polyclonal (PLA)	Proteintech	20566-1-AP	1:100
Anti-Cd68 rabbit polyclonal antibody	Abcam	ab12512	1:100
**Secondary antibodies**			
Goat anti-Rabbit (H+L), Cross-Adsorbed Secondary Antibody, Alexa Fluor 488	Thermo Fisher Scientific	A-11008	1:1000
Goat anti-Mouse (H+L), Cross-Adsorbed Secondary Antibody, Alexa Fluor 594	Thermo Fisher Scientific	A-11032	1:1000

Supplementary Table 3 – List of antibodies used for immunocytochemistry and immunohistochemistry.
