# Supplementary material for: Mitochondrial RNA cytosolic leakage drives the SASP
Source: Nat Commun. 2025 Dec 15;16:10992. doi: 10.1038/s41467-025-66159-z (PMC12705736; doi:10.1038/s41467-025-66159-z)
Supplement: Supplementary file 2 — Reporting Summary [file 41467_2025_66159_MOESM2_ESM.pdf]

nature portfolio

Corresponding author(s): Joao Passos & Stella Victorelli

Last updated by author(s): 10/29/2025

Reporting Summary

Nature Portfolio wishes to improve the reproducibility of the work that we publish. This form provides structure for consistency and transparency in reporting. For further information on Nature Portfolio policies, see our Editorial Policies and the Editorial Policy Checklist.

Statistics

For all statistical analyses, confirm that the following items are present in the figure legend, table legend, main text, or Methods section.

n/a	Confirmed	
☐	☒	The exact sample size (*n*) for each experimental group/condition, given as a discrete number and unit of measurement
☐	☒	A statement on whether measurements were taken from distinct samples or whether the same sample was measured repeatedly
☐	☒	The statistical test(s) used AND whether they are one- or two-sided<br>*Only common tests should be described solely by name; describe more complex techniques in the Methods section.*
☐	☒	A description of all covariates tested
☐	☒	A description of any assumptions or corrections, such as tests of normality and adjustment for multiple comparisons
☐	☒	A full description of the statistical parameters including central tendency (e.g. means) or other basic estimates (e.g. regression coefficient) AND variation (e.g. standard deviation) or associated estimates of uncertainty (e.g. confidence intervals)
☐	☒	For null hypothesis testing, the test statistic (e.g. *F*, *t*, *r*) with confidence intervals, effect sizes, degrees of freedom and *P* value noted<br>*Give P values as exact values whenever suitable.*
☒	☐	For Bayesian analysis, information on the choice of priors and Markov chain Monte Carlo settings
☒	☐	For hierarchical and complex designs, identification of the appropriate level for tests and full reporting of outcomes
☒	☐	Estimates of effect sizes (e.g. Cohen's *d*, Pearson's *r*), indicating how they were calculated

Our web collection on statistics for biologists contains articles on many of the points above.

Software and code

Policy information about availability of computer code

Data collection	CFX Maestro Software, LAS X Office
Data analysis	GraphPad Prism v10, Ingenuity® Pathway Analysis (IPA) software, ImageJ

For manuscripts utilizing custom algorithms or software that are central to the research but not yet described in published literature, software must be made available to editors and reviewers. We strongly encourage code deposition in a community repository (e.g. GitHub). See the Nature Portfolio guidelines for submitting code & software for further information.

Data

Policy information about availability of data

All manuscripts must include a data availability statement. This statement should provide the following information, where applicable:

- Accession codes, unique identifiers, or web links for publicly available datasets
- A description of any restrictions on data availability
- For clinical datasets or third party data, please ensure that the statement adheres to our policy

The RNA-seq datasets generated and analyzed during the current study are available in the GEO repository GSE306839 [https://www.ncbi.nlm.nih.gov/geo/query/acc.cgi?acc=GSE306839] and GSE306957 [https://www.ncbi.nlm.nih.gov/geo/query/acc.cgi?acc=GSE306957]. Source data are provided with this paper.

Research involving human participants, their data, or biological material

Policy information about studies with human participants or human data. See also policy information about sex, gender (identity/presentation), and sexual orientation and race, ethnicity and racism.

Reporting on sex and gender	N/A
Reporting on race, ethnicity, or other socially relevant groupings	N/A
Population characteristics	N/A
Recruitment	N/A
Ethics oversight	N/A

Note that full information on the approval of the study protocol must also be provided in the manuscript.

Field-specific reporting

Please select the one below that is the best fit for your research. If you are not sure, read the appropriate sections before making your selection.

[x] Life sciences [ ] Behavioural & social sciences [ ] Ecological, evolutionary & environmental sciences

For a reference copy of the document with all sections, see nature.com/documents/nr-reporting-summary-flat.pdf

Life sciences study design

All studies must disclose on these points even when the disclosure is negative.

Sample size	For in vivo mouse studies, we have projected group size estimates based on power analyses and previous experiences
Data exclusions	No data was excluded
Replication	We tested our hypothesis using multiple approaches when feasible to enhance scientific rigor and reproducibility. In vitro, all experiments were performed at least 3 times independently.
Randomization	Animals were randomly assigned numbers at weaning. Once assigned to groups, the genotype was not be linked to the numbers until data analysis following completion of all studies.
Blinding	Investigators were blinded to allocation during experiments and outcome assessments, and data was collected and analyzed in a blinded fashion.

Reporting for specific materials, systems and methods

We require information from authors about some types of materials, experimental systems and methods used in many studies. Here, indicate whether each material, system or method listed is relevant to your study. If you are not sure if a list item applies to your research, read the appropriate section before selecting a response.

Materials & experimental systems

n/a	Involved in the study
[ ]	[x] Antibodies
[ ]	[x] Eukaryotic cell lines
[x]	[ ] Palaeontology and archaeology
[ ]	[x] Animals and other organisms
[x]	[ ] Clinical data
[x]	[ ] Dual use research of concern
[x]	[ ] Plants

Methods

n/a	Involved in the study
[x]	[ ] ChIP-seq
[x]	[ ] Flow cytometry
[x]	[ ] MRI-based neuroimaging

Antibodies

Antibodies used	MAVS Santa Cruz SC166583<br>p21 Abcam Ab109199<br>p16 Cell Signalling 92803S<br>MDA5 (IFIH1) Abcam Ab126630

RIG-1 (DDX58) Abcam Ab180675
TLR3 Abcam Ab62566
Actin Sigma A2066
TOM20 Sigma HPA011562
GAPDH Cell Signalling 5174S
PCNA Abcam Ab29
Tubulin Cell Signalling 2146S
BAX Cell Signalling 2772T
BAK Cell Signalling 12105S
HSP90 Cell Signalling 48745
NDUFB8 Abcam Ab110242
UQCRC2 Abcam Ab14745
COX IV Abcam ab33985
Anti-dsRNA J2 mouse monoclonal antibody Jena Bioscience RNT-SCI-10010200
Anti-TOM20 rabbit polyclonal antibody Millipore Sigma HPA011562
Anti-DNA mouse monoclonal antibody Millipore Sigma CBL186
Anti-Phosphorylated Histone H2A.X (S139) (20E3) Cell Signaling 9718S
Anti-MAVS (E-3) mouse monoclonal antibody Santa Cruz Sc-166583
Anti-RIG-1/DDX58 rabbit polyclonal (PLA) Proteintech 20566-1-AP
Anti-Cd68 rabbit polyclonal antibody Abcam ab12512
Goat Anti-Rabbit HRP Conjugated Sigma Aldrich A0545
Goat Anti-Mouse HRP Conjugated Sigma Aldrich A2554
Goat anti-Rabbit (H+L), Cross-Adsorbed Secondary Antibody, Alexa Fluor 488 Thermo Fisher Scientific A-11008
Goat anti-Mouse (H+L), Cross-Adsorbed Secondary Antibody, Alexa Fluor 594 Thermo Fisher Scientific A-11032

Validation

All antibodies used were purchased from commercial sources and reputable vendors (eg. Abcam, Cell Signaling). We selected them based on cross-reactivity
with mouse and human (depending on our research needs) and carefully evaluated the validation data provided by both the vendors and literature.
In several instances, we performed further validation of antibodies by examining the molecular weight of the band by Western blotting and Knocking out protein of
interest. Antibodies were aliquoted and stored as recommended by the manufacturer to minimize freeze thaw cycles and associated loss in performance.

Eukaryotic cell lines

Policy information about cell lines and Sex and Gender in Research

Cell line source(s)	IMR90 and MRC5 fibroblasts were acquired from ATCC
Authentication	none of the cells have been authenticated
Mycoplasma contamination	all cell lines used have been regularly tested for Mycoplasm
Commonly misidentified lines (See ICLAC register)	*Name any commonly misidentified cell lines used in the study and provide a rationale for their use.*

Animals and other research organisms

Policy information about studies involving animals; ARRIVE guidelines recommended for reporting animal research, and Sex and Gender in Research

Laboratory animals	Information regarding experiments involving laboratory mice is in methods section.
Wild animals	*Provide details on animals observed in or captured in the field; report species and age where possible. Describe how animals were caught and transported and what happened to captive animals after the study (if killed, explain why and describe method; if released, say where and when) OR state that the study did not involve wild animals.*
Reporting on sex	Only male mice were used. All animal experiments using MASH animal models were conducted in male mice, as female mice are well known not to develop the diet-induced metabolic changes characteristic of the disease. This is clearly specified in methods and figure legends.
Field-collected samples	*For laboratory work with field-collected samples, describe all relevant parameters such as housing, maintenance, temperature, photoperiod and end-of-experiment protocol OR state that the study did not involve samples collected from the field.*
Ethics oversight	All animal experiments were performed according to protocols approved by the Institutional Animal Care and Use Committee (IACUC) at Mayo Clinic.

Note that full information on the approval of the study protocol must also be provided in the manuscript.

Plants

Seed stocks	*Report on the source of all seed stocks or other plant material used. If applicable, state the seed stock centre and catalogue number. If plant specimens were collected from the field, describe the collection location, date and sampling procedures.*
Novel plant genotypes	*Describe the methods by which all novel plant genotypes were produced. This includes those generated by transgenic approaches, gene editing, chemical/radiation-based mutagenesis and hybridization. For transgenic lines, describe the transformation method, the number of independent lines analyzed and the generation upon which experiments were performed. For gene-edited lines, describe the editor used, the endogenous sequence targeted for editing, the targeting guide RNA sequence (if applicable) and how the editor was applied.*
Authentication	*Describe any authentication procedures for each seed stock used or novel genotype generated. Describe any experiments used to assess the effect of a mutation and, where applicable, how potential secondary effects (e.g. second site T-DNA insertions, mosiacism, off-target gene editing) were examined.*
