# Supplementary material for: Mitochondrial RNA cytosolic leakage drives the SASP
Source: Nat Commun. 2025 Dec 15;16:10992. doi: 10.1038/s41467-025-66159-z (PMC12705736; doi:10.1038/s41467-025-66159-z)
Supplement: Supplementary file 4 — Source data [file 41467_2025_66159_MOESM4_ESM.zip › VictorelliSD/Original WB images_compressed.pptx]

## Slide 1
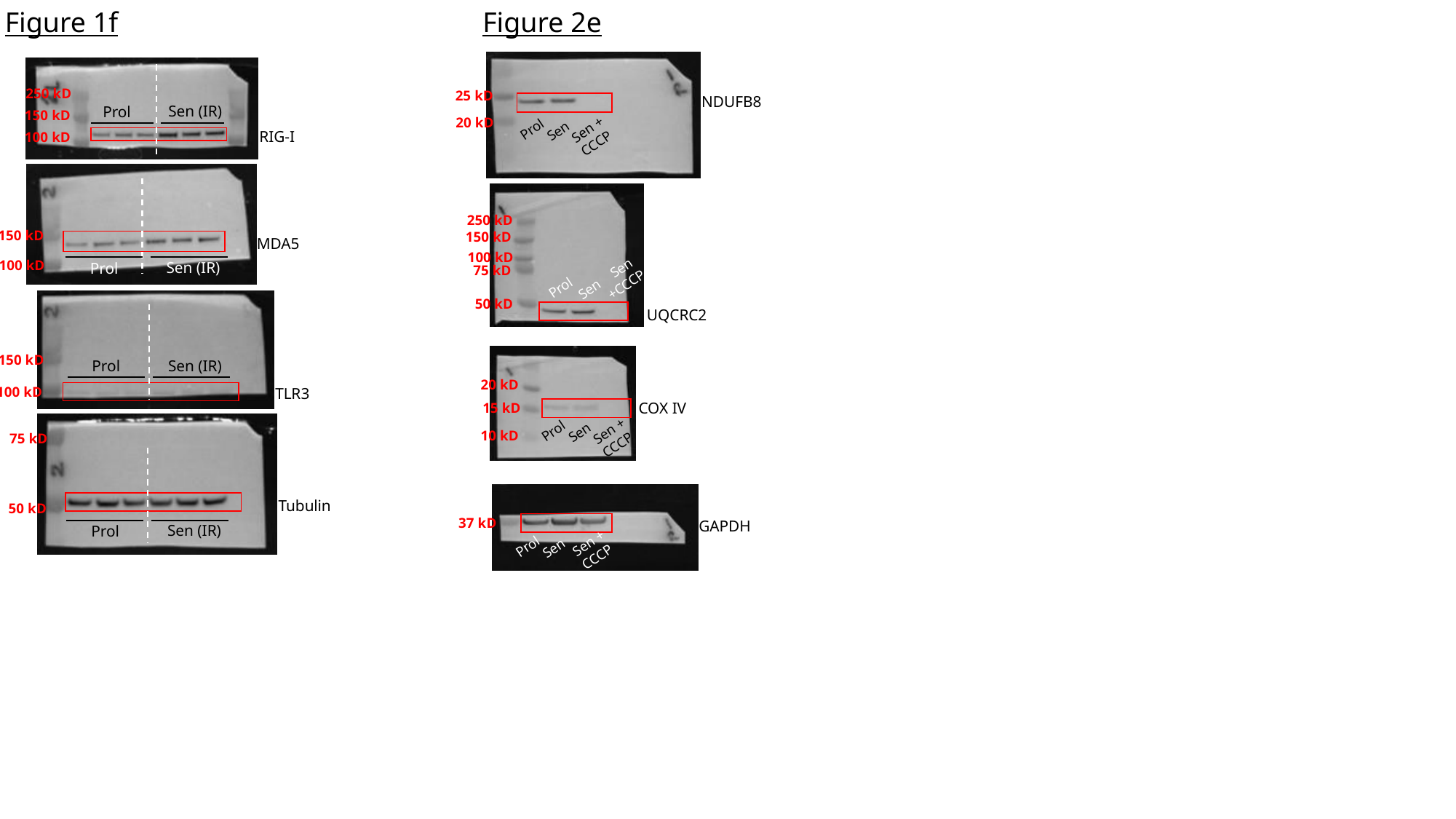

Figure 1f
Figure 2e
250 kD
25 kD
NDUFB8
Sen (IR)
Prol
150 kD
20 kD
Sen +
CCCP
Prol
Sen
RIG-I
100 kD
250 kD
150 kD
150 kD
MDA5
100 kD
100 kD
Sen (IR)
Prol
75 kD
Sen
+CCCP
Prol
Sen
50 kD
UQCRC2
150 kD
Sen (IR)
Prol
20 kD
100 kD
TLR3
COX IV
15 kD
Sen +
CCCP
Prol
Sen
10 kD
75 kD
Tubulin
50 kD
37 kD
GAPDH
Sen (IR)
Prol
Sen +
CCCP
Prol
Sen

## Slide 2
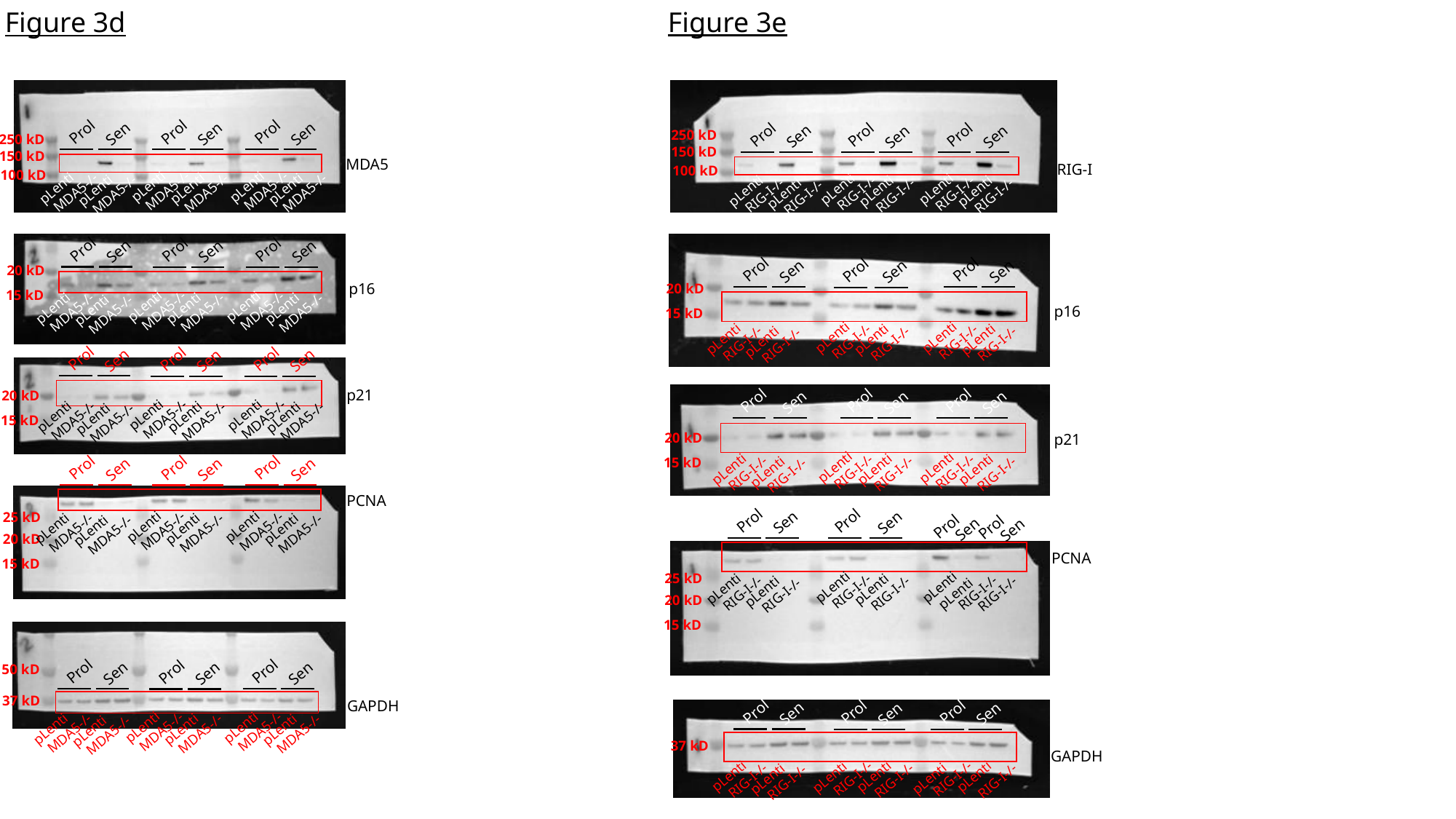

Figure 3d
Figure 3e
Prol
Prol
Prol
Sen
Sen
Sen
Prol
Prol
Prol
250 kD
Sen
Sen
Sen
250 kD
150 kD
150 kD
MDA5
RIG-I
100 kD
100 kD
pLenti
pLenti
pLenti
pLenti
pLenti
pLenti
pLenti
pLenti
pLenti
MDA5-/-
pLenti
MDA5-/-
pLenti
MDA5-/-
MDA5-/-
MDA5-/-
RIG-I-/-
RIG-I-/-
pLenti
MDA5-/-
RIG-I-/-
RIG-I-/-
RIG-I-/-
RIG-I-/-
Prol
Prol
Prol
Sen
Sen
Sen
Prol
Prol
Prol
20 kD
Sen
Sen
Sen
p16
20 kD
15 kD
pLenti
pLenti
pLenti
pLenti
pLenti
pLenti
p16
MDA5-/-
MDA5-/-
MDA5-/-
MDA5-/-
MDA5-/-
15 kD
MDA5-/-
pLenti
pLenti
pLenti
pLenti
pLenti
RIG-I-/-
RIG-I-/-
pLenti
RIG-I-/-
RIG-I-/-
RIG-I-/-
RIG-I-/-
Prol
Prol
Prol
Sen
Sen
Sen
p21
20 kD
Prol
Prol
Prol
Sen
Sen
Sen
pLenti
pLenti
pLenti
pLenti
pLenti
pLenti
MDA5-/-
MDA5-/-
15 kD
MDA5-/-
MDA5-/-
MDA5-/-
MDA5-/-
20 kD
p21
15 kD
Prol
Prol
Prol
pLenti
pLenti
Sen
Sen
Sen
pLenti
pLenti
pLenti
RIG-I-/-
RIG-I-/-
pLenti
RIG-I-/-
RIG-I-/-
RIG-I-/-
RIG-I-/-
PCNA
25 kD
Prol
Prol
Sen
Sen
Prol
Prol
pLenti
pLenti
Sen
pLenti
pLenti
Sen
pLenti
pLenti
MDA5-/-
MDA5-/-
MDA5-/-
MDA5-/-
MDA5-/-
MDA5-/-
20 kD
PCNA
15 kD
25 kD
pLenti
pLenti
pLenti
pLenti
RIG-I-/-
pLenti
RIG-I-/-
RIG-I-/-
RIG-I-/-
pLenti
RIG-I-/-
RIG-I-/-
20 kD
15 kD
50 kD
Prol
Prol
Prol
Sen
Sen
Sen
37 kD
GAPDH
Prol
Prol
Prol
Sen
Sen
Sen
pLenti
pLenti
pLenti
pLenti
pLenti
pLenti
MDA5-/-
MDA5-/-
MDA5-/-
MDA5-/-
MDA5-/-
MDA5-/-
37 kD
GAPDH
pLenti
pLenti
pLenti
pLenti
RIG-I-/-
pLenti
RIG-I-/-
pLenti
RIG-I-/-
RIG-I-/-
RIG-I-/-
RIG-I-/-

## Slide 3
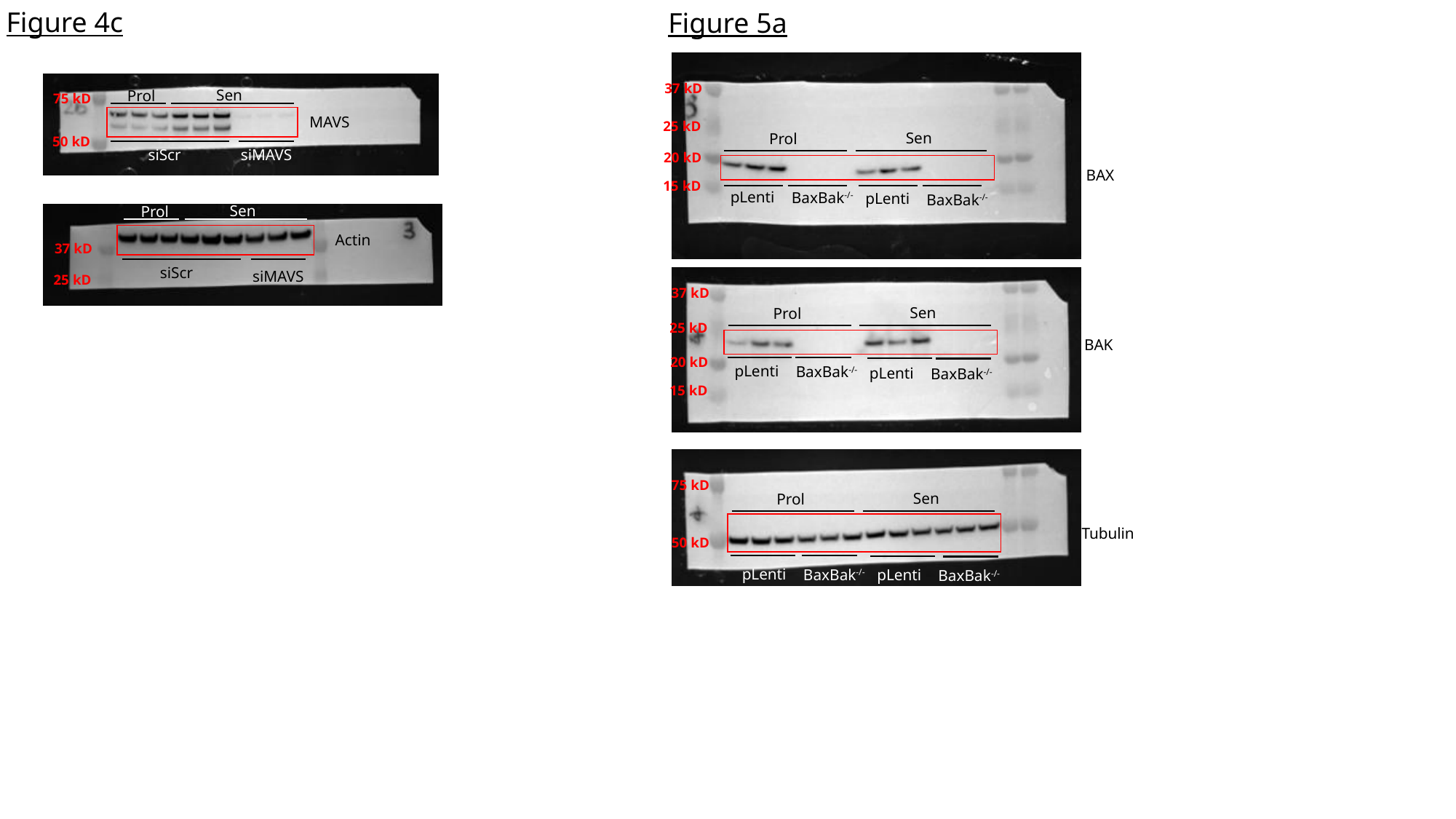

Figure 4c
Figure 5a
37 kD
Sen
Prol
75 kD
MAVS
25 kD
Sen
Prol
50 kD
siScr
siMAVS
20 kD
BAX
15 kD
pLenti
BaxBak-/-
pLenti
BaxBak-/-
Sen
Prol
Actin
37 kD
siScr
siMAVS
25 kD
37 kD
Sen
Prol
25 kD
BAK
20 kD
pLenti
BaxBak-/-
pLenti
BaxBak-/-
15 kD
75 kD
Sen
Prol
Tubulin
50 kD
pLenti
pLenti
BaxBak-/-
BaxBak-/-

## Slide 4
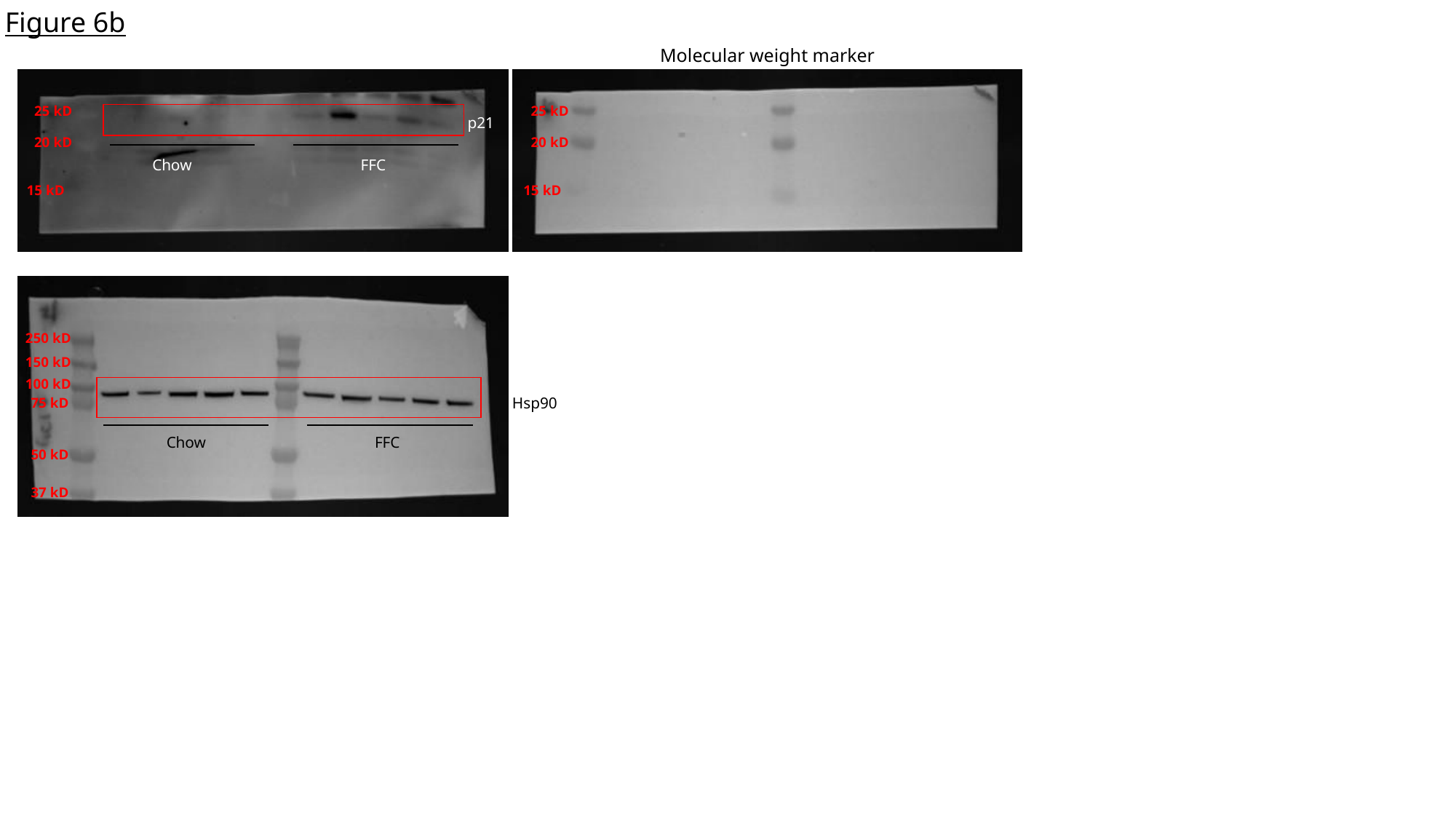

Figure 6b
Molecular weight marker
25 kD
25 kD
p21
20 kD
20 kD
Chow
FFC
15 kD
15 kD
250 kD
150 kD
100 kD
Hsp90
75 kD
Chow
FFC
50 kD
37 kD

## Slide 5
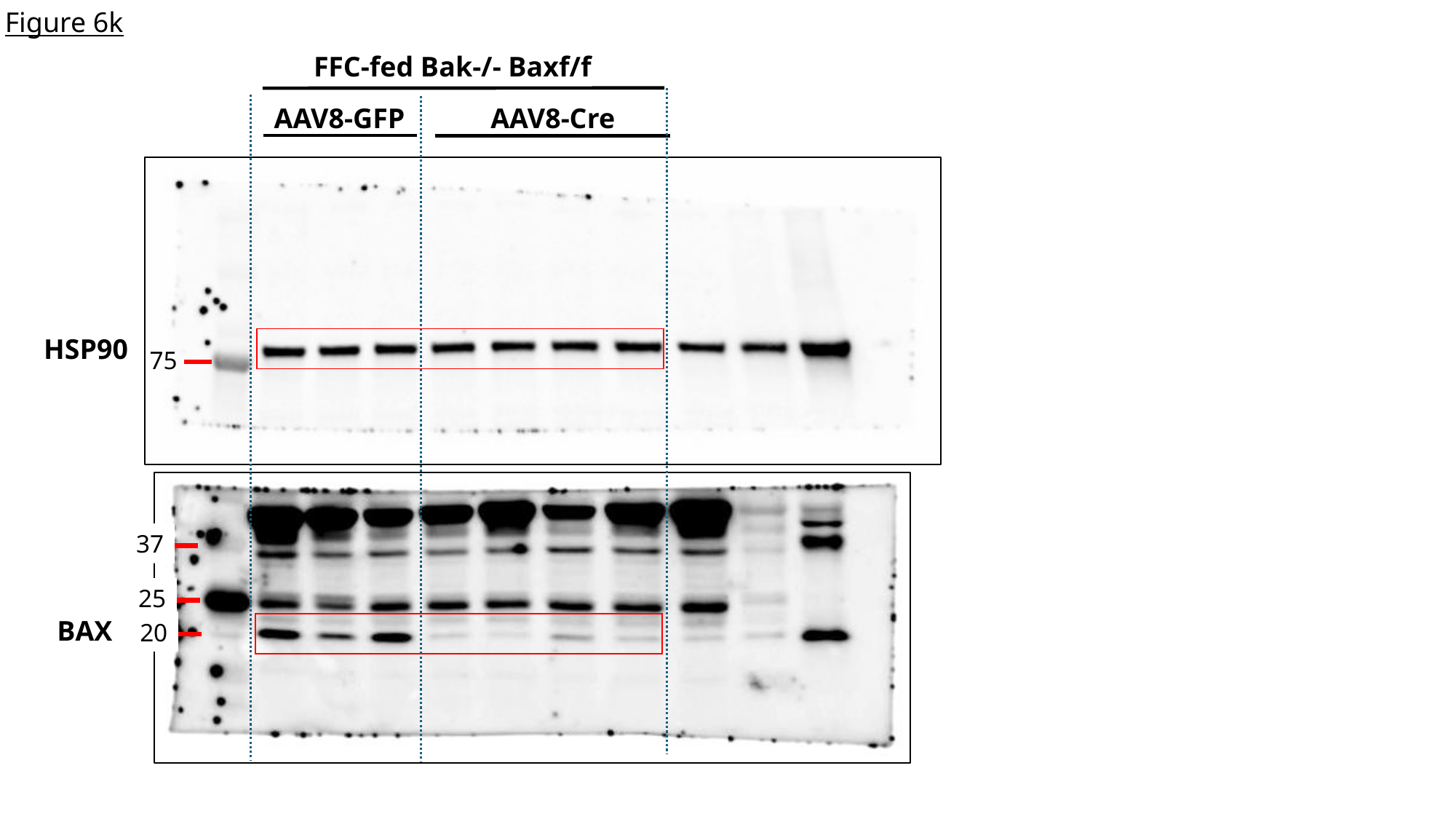

Figure 6k
FFC-fed Bak-/- Baxf/f
AAV8-Cre
AAV8-GFP
HSP90
75
37
25
BAX
20

## Slide 6
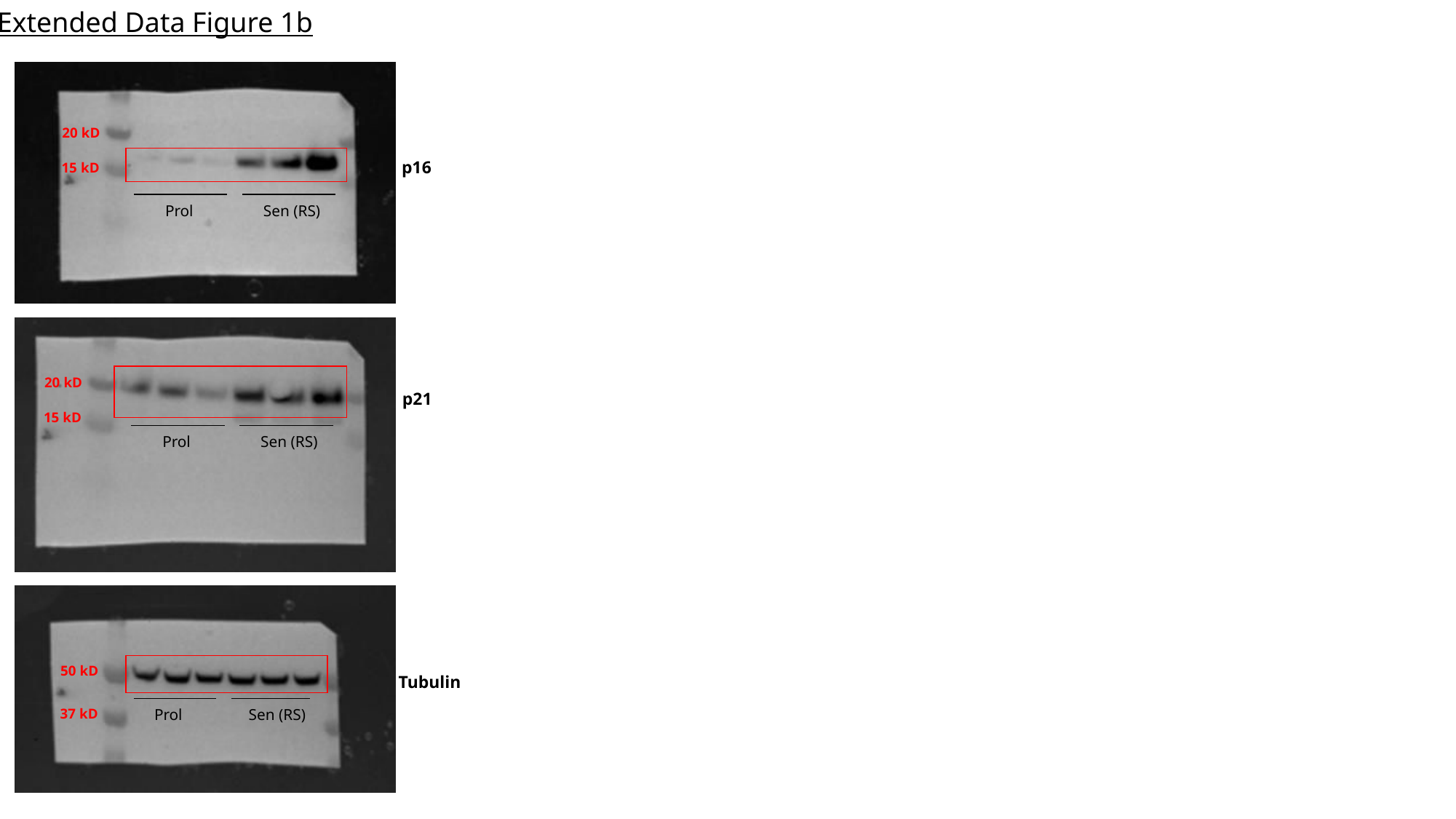

Extended Data Figure 1b
20 kD
p16
15 kD
Prol
Sen (RS)
20 kD
p21
15 kD
Prol
Sen (RS)
50 kD
Tubulin
37 kD
Prol
Sen (RS)

## Slide 7
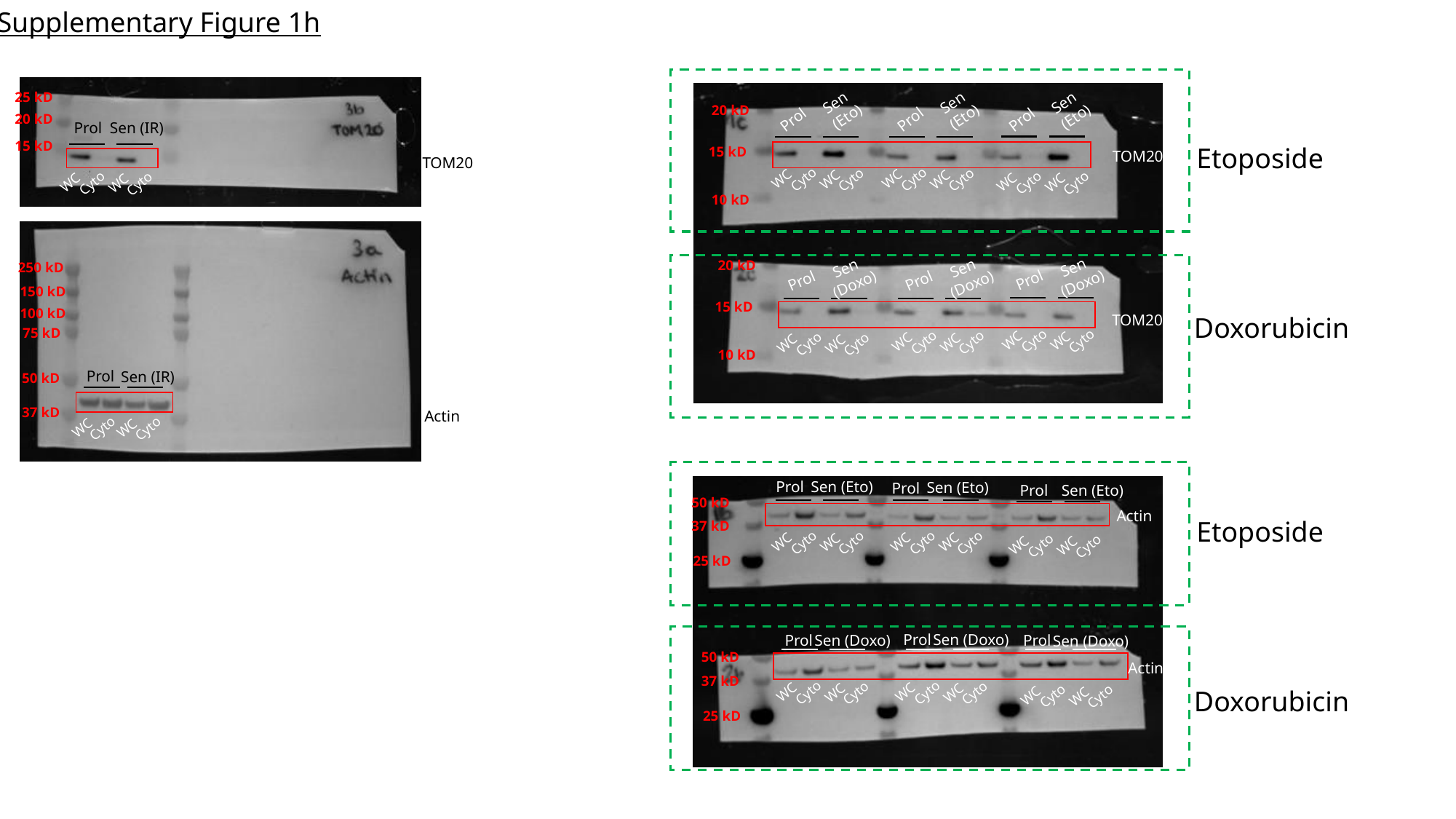

Supplementary Figure 1h
25 kD
Sen
(Eto)
Sen
(Eto)
Sen
(Eto)
20 kD
Prol
Prol
Prol
20 kD
Prol
Sen (IR)
15 kD
Etoposide
15 kD
TOM20
TOM20
WC
WC
Cyto
Cyto
WC
WC
Cyto
Cyto
WC
Cyto
WC
WC
Cyto
Cyto
WC
Cyto
10 kD
Sen
(Doxo)
20 kD
Sen
(Doxo)
Sen
(Doxo)
250 kD
Prol
Prol
Prol
150 kD
15 kD
100 kD
TOM20
Doxorubicin
75 kD
WC
Cyto
WC
Cyto
WC
Cyto
WC
Cyto
WC
Cyto
WC
Cyto
10 kD
Prol
Sen (IR)
50 kD
37 kD
Actin
WC
Cyto
WC
Cyto
Sen (Eto)
Prol
Sen (Eto)
Prol
Prol
Sen (Eto)
50 kD
Actin
Etoposide
37 kD
WC
WC
Cyto
Cyto
WC
WC
Cyto
Cyto
WC
Cyto
WC
Cyto
25 kD
Prol
Sen (Doxo)
Prol
Sen (Doxo)
Prol
Sen (Doxo)
50 kD
Actin
37 kD
WC
WC
Cyto
Cyto
WC
WC
Cyto
Cyto
Doxorubicin
WC
Cyto
WC
Cyto
25 kD

## Slide 8
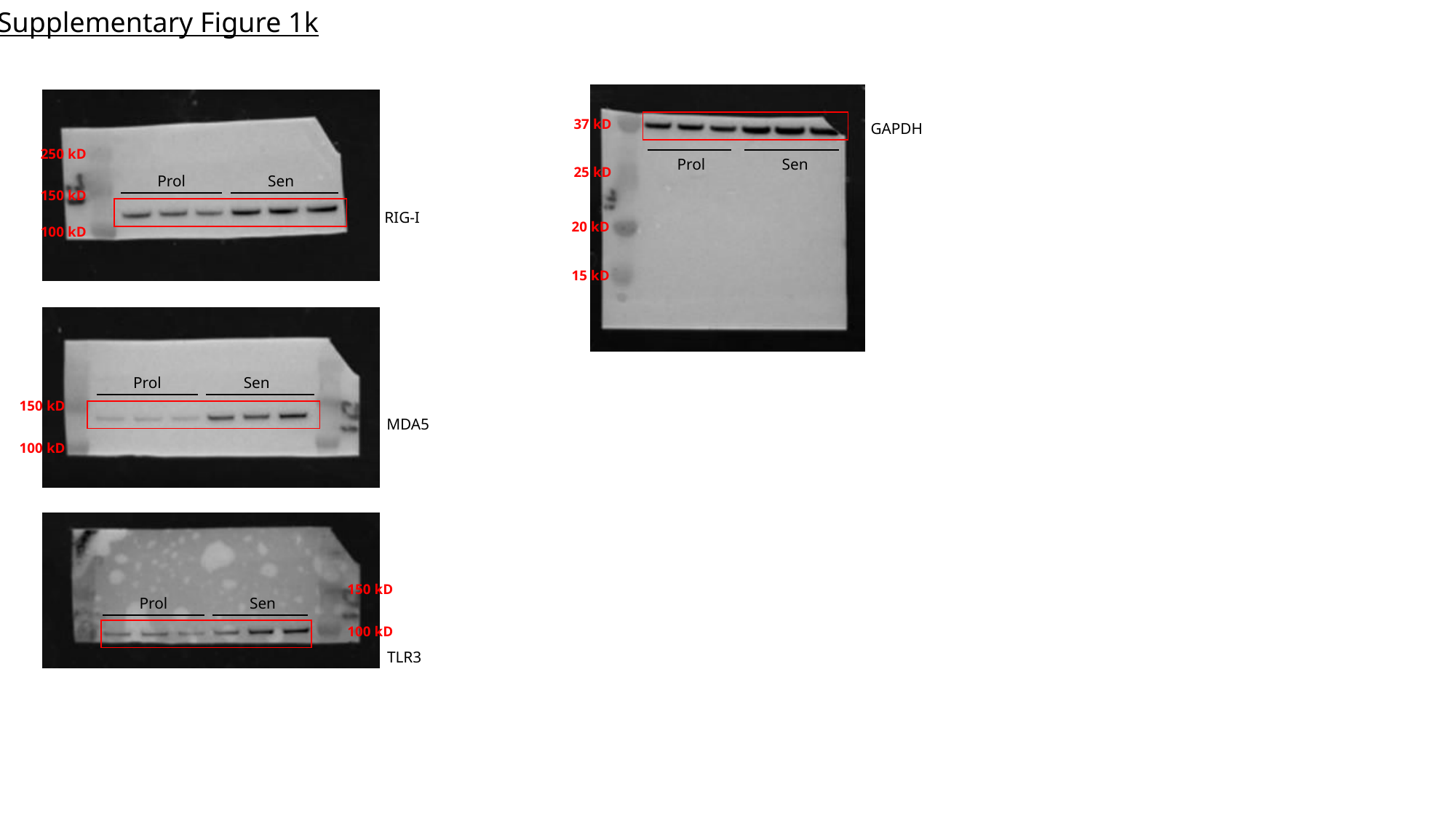

Supplementary Figure 1k
37 kD
GAPDH
250 kD
Prol
Sen
25 kD
Prol
Sen
150 kD
RIG-I
20 kD
100 kD
15 kD
Prol
Sen
150 kD
MDA5
100 kD
150 kD
Prol
Sen
100 kD
TLR3
